# Comparative chloroplast genomes: insights into the identification and phylogeny of rapid radiation genus *Rhodiola*

**DOI:** 10.3389/fpls.2024.1404447

**Published:** 2024-05-10

**Authors:** Jinxin Liu, Erhuan Zang, Yu Tian, Liqiu Zhang, Yimin Li, Linchun Shi, Lijia Xu, Peigen Xiao

**Affiliations:** ^1^ State Key Laboratory for Quality Ensurance and Sustainable Use of Dao-di Herbs, Institute of Medicinal Plant Development, Chinese Academy of Medical Sciences and Peking Union Medical College, Beijing, China; ^2^ Engineering Research Center of Chinese Medicine Resource, Ministry of Education, Beijing, China; ^3^ Hebei Key Laboratory of Study and Exploitation of Chinese Medicine, Chengde Medical University, Chengde, China; ^4^ School of Medicine and Pharmacy, Tonghua Normal University, Tonghua, China; ^5^ College of Pharmacy and Key Laboratory for Research and Development of “Qin Medicine” of Shaanxi Administration of Chinese Medicine, Shaanxi University of Chinese Medicine, Xianyang, China

**Keywords:** *Rhodiola*, chloroplast genomes, plastomes, rapid radiation, phylogenetic relationship

## Abstract

*Rhodiola* L. is a genus exhibiting rapid radiation and represents a typical case for studying plastid gene adaptation in species that spread from high altitudes to low altitudes. In this study, 23 samples of 18 *Rhodiola* species were collected from the Qinghai-Tibetan Plateau and five scattered alpine areas, and the plastid genomes (plastomes) of these species were sequenced, annotated, and compared between high-altitude and widely distributed groups. The plastomes of *Rhodiola* were found to be highly conserved in terms of gene size, content, and order but highly variable in several lineage-specific features, such as codon usage bias, IR boundary shifting, and distinct repeat sequence structures binding to SSRs. Codon usage in the genes of photosystem II exhibited an obvious preference, reflecting significant environmental adaptation pressures. In this study, three repeat regions compounded with trinucleotide and mononucleotide repeats were found for the first time in *R. forrestii*, *R. himalensis*, and *R. yunnanensis*. High-variability regions such as *ndhF*, *ycf1*, *trnH-psbA*, and *rpoC1-rpoB* were screened, laying the foundation for the precise identification of these species. The phylogenetic analysis revealed the occurrence of cyto-nuclear discordance, likely originating from the frequent interspecific hybridization events observed within *Rhodiola* species during rapid radiation. Dioecious and hermaphrodite species can be broadly categorized into two subclades, probably they have different environmental adaptation strategies in response to climate change. In addition, the phylogenetic tree supported the monophyly of *R. forrestii* and *R. yunnanensis*, which compose *R.* Sect*. Pseudorhodiola*. In conclusion, plastome data enrich the genetic information available for the *Rhodiola* genus and may provide insight into species migration events during climate change.

## 1 Introduction

*Rhodiola* L. is a perennial herbaceous genus that belongs to the family Crassulaceae and has approximately 90 species worldwide (Fu and Ohba, 2001). Most *Rhodiola* species grow on slopes or rock outcrops of limestones and granites in the cold regions of the Northern Hemisphere (Fu and Ohba, 2001). *Rhodiola* plants have evolved several specific morphological traits to facilitate its radiation in high-altitude regions (Zhu et al., 2022), characterized by high cold, hypoxia, strong UV radiation, and strong winds with sand. For example, some species exhibit dimorphism, elongated rhizomes, marcescent old flower stems, or scaly cauline leaves (Ren et al., 2023). Many species, such as *R. rosea*, *R. crenulata*, and *R. sacra*, are used as medicinal herbs in China, Russia, Mongolia, and America. Currently, approximately 300 constituents have been identified in these *Rhodiola* plants (Zhou et al., 2014), which are rich in polyphenols, salidroside and tyrosol. The pharmacological effects (Chiang et al., 2015; Zhuang et al., 2019; Pu et al., 2020) of these compounds include increasing longevity, stimulating the central nervous system, and exhibiting anti-aging, anti-inflammatory, and antibacterial effects. According to *Flora of China*, there are 55 species (16 endemic) in China (Zhang et al., 2015), mainly distributed on the Qinghai-Tibetan Plateau (QTP) and adjacent areas, at elevations ranging from 3500–5000 m, except for several species distributed in mountainous areas of northwest, northeast, and central China, at elevations of about 1000–2500 m. For example, *R. angusta* and *R. sachalinensis* are restricted to the Changbai Mountain areas at altitudes of 1600–2500 m. *Rhodiola rosea* is distributed in Hebei, Jilin, Shanxi, and Xinjiang provinces of China, as well as in other regions, including Japan, Kazakhstan, Korea, Mongolia, Russia, Europe, and North America. Ancestral state reconstruction supported that *Rhodiola* originated on the QTP (21 Mya) and then dispersed to other regions of the Northern Hemisphere (12 Mya), promoted by the uplifts of the Qinghai-Tibetan Plateau (Zhang et al., 2014b). Therefore, how did species such as *R. rosea* and *R. dumulosa* spread to other unconnected mid- and high-altitude mountains? One study of *R. dumulosa* suggested that this process was accomplished through two migration steps (Hou and Lou, 2014). With the continual increase in temperature, *R. dumulosa* initially migrated from refuges on the QTP to northern and central China through the intervening highlands and subsequently moved even higher toward the mountain peaks of these regions owing to its preference for cooler climates.

*Rhodiola* is a genus with rapid radiation, and its exact intergeneric and intrageneric relationships have not yet been fully resolved (Zhang et al., 2014b). This may be due to factors such as introgression, incomplete lineage sorting, and reticulate evolution (You et al., 2021). The genus was initially established by Linnaeus in 1573 but was later reduced to being a synonym of the genus *Sedum*. However, in 2004, phylogenetic analysis of *trnL-trnF* and ITS indicated that *Rhodiola* can be clearly separated from *Sedum*, but it is closely related to *Pseudosedum* because of the traits of bract scales on the flowering stem (Mayuzumi and Ohba, 2004). In 2006, phylogenetic relationships inferred from ITS revealed that *Pseudosedum* was nested within *Rhodiola* (Gontcharova et al., 2006), which has been verified by ITS and eight plastid fragments (Zhang et al., 2014b). With the development of high-throughput sequencing, obtaining chloroplast genomes (plastomes) has become a simple and cost-effective means for phylogenetic and phylogeographic studies (Liu et al., 2012), especially in conjunction with the increasingly sophisticated plastome annotation tools (Shi et al., 2019). In comparison to traditional phylogenetic markers, plastomes can provide detailed information that provides us with the opportunity to gain insights into rapid radiation clades (Chang et al., 2021).

Like most angiosperm groups, the plastomes of *Rhodiola* exhibit a conserved genome structure and gene content. Analyses of positive selection have confirmed that certain genes (*rpl16*, *ndhA*, *ndhH*, and *psaA*) in *Rhodiola* plastomes may have undergone adaptive evolution to thrive in alpine habitats (Xu et al., 2020). Plastome data could significantly improve the ability to reconstruct robust phylogenetic backbones of *Rhodiola* and shed new light on the divergence between hermaphrodite and dioecious groups (Zhao et al., 2021). Therefore, it is crucial to gather more plastome data to resolve the phylogenetic relationships within the infrageneric groups of *Rhodiola*. In this study, we have assembled and annotated the complete plastomes of 23 *Rhodiola* samples from 18 species, attempting to discuss the structural features, codon usage bias, repeat sequences, comparative genomics, and phylogenetic relationships of the infrageneric groups in *Rhodiola*.

## 2 Materials and methods

### 2.1 Taxon sampling, DNA extraction, and high-throughput sequencing

Twenty-three samples from 18 *Rhodiola* species were collected across the Tibetan Plateau and adjacent areas, as well as from the Taibai Mountains, Changbai Mountains, Bashang Plateau, Helan Mountains, and other areas. Yulin Lin, Yaodong Qi, and Xinlei Zhao from the Institute of Medicinal Plant Development, Chinese Academy of Medical Sciences and Peking Union Medical College, along with Jun Zhang from Yunnan Nationalities University, conducted the authentication of these species. Additionally, the plastomes of 36 samples were obtained from *Rhodiola* related research conducted by prominent teams, as well as from professional plant journals (Zhao et al., 2020). In total, our study encompassed 59 *Rhodiola* plastomes, representing three subgenera and six sections of the genus. Furthermore, six species from other genera of the Crassulaceae family were selected as outgroups according to previous studies.

The samples were dried in silica gel in the field or frozen fresh with liquid nitrogen. Genomic DNA was extracted from 20–30 mg of dried tissues or 50–60 mg of frozen tissues using modified CTAB methods according to our previous study. DNA quality was determined by a Qubit 4.0 fluorometer, and the DNA was then fragmented to construct 350 bp PCR-free libraries. Subsequently, these libraries were sequenced using the Illumina NovaSeq platform, and approximately 6 Gb paired end reads with a length of 150 bp were generated for each sample. The raw sequencing reads were trimmed of adapters and low-quality reads using Trimmomatic v0.39 (Bolger et al., 2014), after which paired reads were retained and incorporated into subsequent analysis pipelines.

### 2.2 Plastome assembly, gene annotation, and genome characterization

Plastomes were *de novo* assembled using GetOrganelle v1.7.7.0 (Jin et al., 2020). with default parameters, and their genome mapping statistic was accomplished by reads mapping using Bowtie2 (Langmead and Salzberg, 2012). SAM file output from Bowtie2 was subsequently used to visualize the mapping coverage *via* BRIG (Alikhan et al., 2011) and determine junction regions. Then, the starting point and LSC direction of these initial assembly results were adjusted using our local python script with the plastome of *R. rosea* as the reference (GenBank accession number MH410216). Genes were predicted and annotated by our previously reported tool, CPGAVAS2 (Shi et al., 2019). Gene model generated from CPGAVAS2 were manually curated by Apollo software (Lewis et al., 2002) with supporting information generated by BLAST again reference plastomes deposited in NCBI and Swiss-Prot database. The final annotation results were updated and converted into GenBank format for genome visualization using OGDRAW v1.3.1 (Lohse et al., 2013).

Relative synonymous codon usage (RSCU) and GC content in third positions (GC3) was calculated by CodonW (http://codonw.sourceforge.net). The codon adaptation index (CAI) was calculated with the method of Sharp and Li (1987). The effective number of codons (ENC) for each genome was estimated with the method of Novembre (2002). The predicted curve of ENC and GC3 was plotted with the equation of Wright’s equation (Wright, 1990). Simple sequence repeats (SSRs) analysis was conducted with MISA v2.1 (Beier et al., 2017). Repetitive features such as forward (F), palindromic (P), reverse (R), and complement (C) repeats were identified with the online tool REPuter (https://bibiserv.cebitec.uni-bielefeld.de/reputer). Tandem repeat arrays were detected using Tandem Repeat Finder (https://tandem.bu.edu/trf/trf.html). The GC content for whole genome LSC, SSC, and IR regions was calculated with our local python program.

### 2.3 Plastome comparative genomic analysis and phylogenetic analysis

Nucleotide polymorphism analysis for plastid gene and gene-intergenic regions were estimated by DnaSP v6 (Rozas et al., 2017). Gene rearrangement events were determined and visualized using Mauve v2.3.1 (Darling et al., 2010). In addition, the contraction and extension of IR boundaries between the four parts of the genome (LSC-IRb-SSC-IRa) were visualized by IRScope (https://irscope.shinyapps.io/irapp/). Protein-coding DNA alignment was carried out with ParaAT v2.0 (Zhang et al., 2012).

Two datasets were constructed for phylogenetic analysis: *Rhodiola* samples collected by this study plus outgroup species (Ra), All *Rhodiola* species collected by this study, download from previous studies, as well as outgroup species (Rb). To reconstruct the phylogeny of *Rhoidola* species, multiple sequence alignment of Plastid-coding genes was completed in MAFFT v7 (Katoh and Standley, 2013), and then refined manually in BioEdit v7.2 (Hall, 1999) for ambiguous regions. The maximum likelihood (ML) method was applied for the Ra and Rb datasets using IQ-tree v 2.2.2.6 (Trifinopoulos et al., 2016). Bootstrap values were obtained using ultrafast bootstrap approximation (Hoang et al., 2017) with 1000 replicates. ITS, *matK*, *rbcL*, and *psbA* sequences were assembled using GetOrganelle v1.7.7.0 based on high-throughput sequencing data. The outgroup sequences were downloaded from the NCBI database. The ML method was applied using IQ-tree v 2.2.2.6 software to construct the phylogenetic tree of the Ra dataset, and the bootstrap values were 1000 replicates.

## 3 Results

### 3.1 Genome structure and gene content of *Rhodiola* plastomes

A total of 23 complete plastomes from 18 *Rhodiola* species were sequenced and annotated. The GenBank accession numbers, Biosample and SRA information for these plastomes were presented in **Supplementary Table 1**. The length of these plastomes is ranged from 150,893 bp to 151,946 bp, with variations primarily attributed to insertions and deletions within non-coding regions. For example, regions like *psbA-trnH* exhibit numerous “TATA” repeat units of varying lengths, contributing significantly to the length discrepancies. Each plastome newly sequenced in this study has a typical four-part structure, including a large single copy (LSC) region (82,195–83,149 bp), a small single copy (SSC) region (16,873–17,117 bp) and a pair of reverse repeats (IR) regions (IRa and IRb) (25,690–25,956 bp). The GC content of complete plastomes ranged from 37.68% to 37.78%, and the IR regions (about 43%) was higher than the LSC region and SSC region (about 36% and 32%).

**Figure 1:**
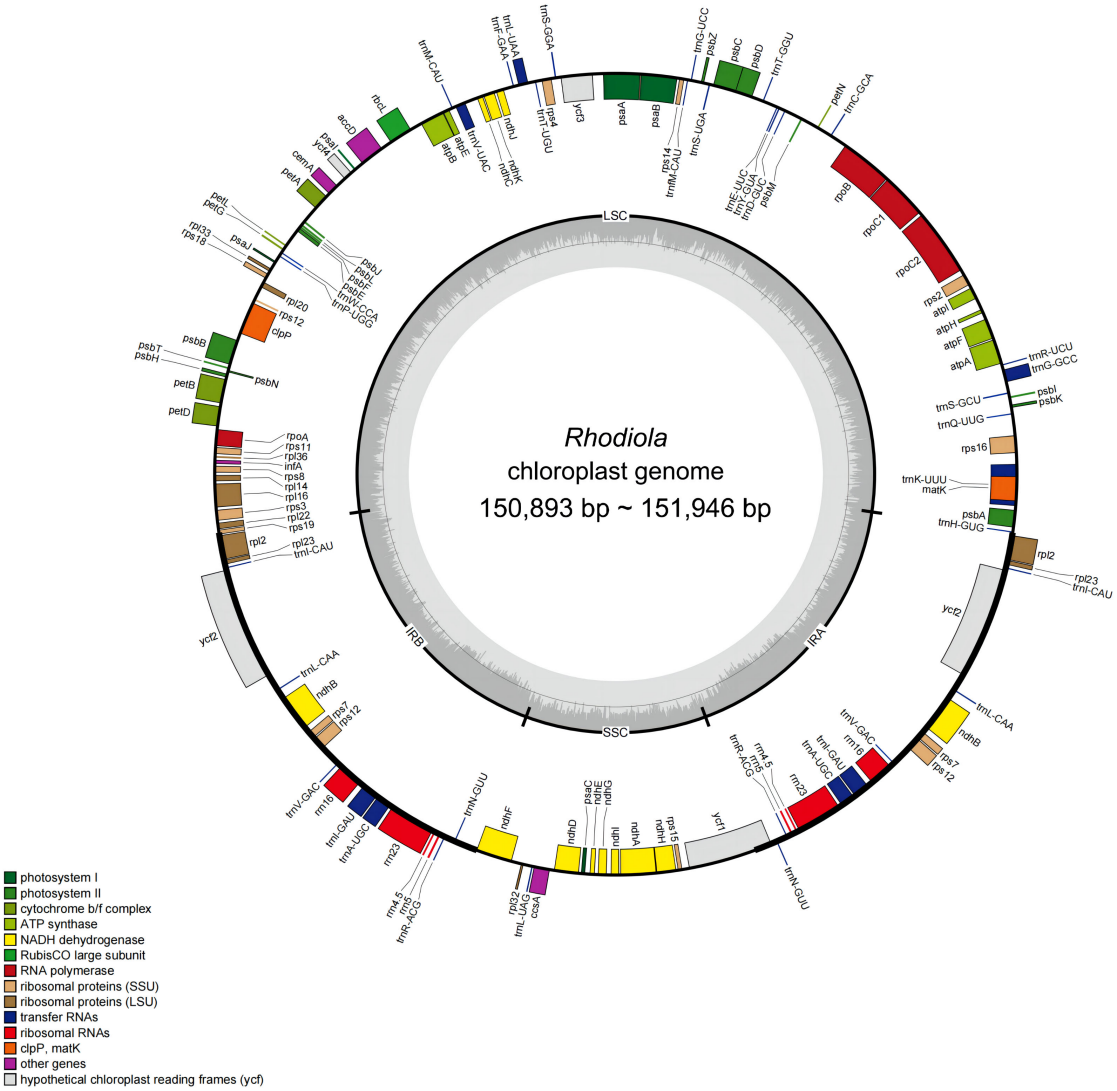
Gene map of 23 complete plastomes from 18 *Rhodiola* species. The genes drawn outside the circle are transcribed clockwise, while those inside the circle are transcribed counterclockwise. Genes are color coded by functional group. The LSC (large single copy region), SSC (small single copy region), and the IRA and IRB (inverted repeats) are displayed on the inner circle, with GC content in dark gray and AT content in light gray.

*Rhodiola* plastomes encodes same gene content and gene number. A total of 113 unique genes were annotated for each plastome, including 79 protein-coding genes, 30 tRNA genes, and 4 rRNA genes (*rrn23S*, *rrn16S*, *rrn5S*, and *rrn4.5S*). Eighteen genes are in the IR regions, of which one copy of *rsp19* and *ycf1* is a potential pseudogene. In addition, 18 genes contained introns: 15 genes (nine protein-coding genes and six tRNA genes) contained one intron, and three genes (*rps12, ycf3*, and *clpP*) contained two introns. Small exons were also annotated in the *petB*, *petD*, and *rpl16* genes, with lengths of 6 bp, 8 bp, and 9 bp, respectively. Finally, *rps12* was identified as a trans-splicing gene. The genome structure and gene content of *Rhodiola* were shown in **Figure 1** and **Supplementary Table 2**.

### 3.2 Codon usage analysis

**Figure 2:**
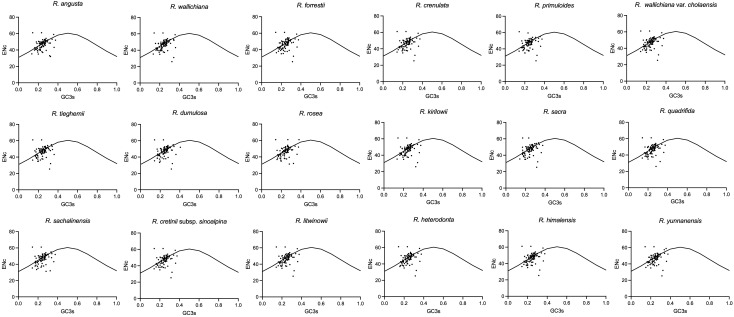
The ENc vs GC3 plots of *Rhodiola* plastomes. The standard curves (ENc = 2 + GC3 + 29/[GC3^2^ + (1 − GC3)^2^]) in the figures are the expected values of ENc to GC3.

In codon analysis, the average number of codons for *Rhodiola* species is 22,673. Among them, codons encoding leucine were the most abundant amino acid codons, with a frequency of 10.77%, whereas those used to encode cysteine were the scarcest amino acid codons, at 1.1%. AUU (encoding isoleucine) and UGC (encoding cysteine) were the most and least used codons, respectively. Thirty codons had RSCU values greater than 1, 32 codons had RSCU values less than 1, and only two codons (AUG and UGG) had RSCU values equal to 1. Among all the genes analyzed, the CAI values ranged from 0.092 to 0.305, and the gene with the lowest CAI value was *rpl20*. Interestingly, ENc values varied widely across genes, ranging from 23.13 (*petN*) to 52.06 (*ycf2*). *rpl16*, *psbM* and *petN* had the lowest GC3 content, which were 0.1757, 0.1808 and 0.2077, respectively. In contrast, the GC3 values of *psbT*, *ycf2* and *rpl36* are as high as 0.4825, 0.4678 and 0.4653, respectively. In addition, except for methionine and tryptophan, almost all amino acids have multiple synonymous codons, of which arginase, leucine, and serine have six synonymous codons. Five types of start codons were detected in 79 protein-coding genes. Among them, 74 genes used AUG as the start codon, one gene (*psbL*) used ACG, two genes (*rps19* and *ycf2*) used GUG, and the *ndhH* and *ndhK* genes used ATC and ATT as start codons, respectively. UAA, UAG, and UGA are the stop codons of these genes. The most frequently used stop codon was UAA (53.16%), followed by UGA (24.21%) and UAG (22.63%). To analyze changes in codon usage and nucleotide composition, scatter plots of ENC versus GC3 were generated, and Wright’s standard equations were superimposed on each plot (**Figure 2**). Most of the genes were found to be above and below the equation (Wright, 1990; Zhang et al., 2018a). Detailed information on the codon suage analysis can be found in **Supplementary Figure 1** and **Supplementary Table 3**.

### 3.3 Repeat elements in *Rhodiola* plastomes

The repeat elements, such as SSRs, tandem repeats, dispersed repeats and palindromic repeats were analyzed. The number of SSRs among different species varied from 48 to 58, and the species with the greatest number of SSRs was *R. yunnanensis*. Except for mono- to hex- repeats, three repeat regions compounded with trinucleotide repeats and monomeric repeats were found in *R. forrestii*, *R. himalensis*, and *R. yunnanensis*, which can be used as a species-identifying features (**Supplementary Table 4**). There are 124 unique tandem repeat patterns in total, of which 66 patterns appear only once in a specific plastome, and four patterns (“TTTGTCTAAGTCACTTCTCTT”, “TAGTGATCATTTATC”, “GATAAATAATCACTA”, “AAAAAGAGAAGTGACTTAGAC”) can be found in all plastomes. The average number of tandem repeats pattens for each plastome is 20.14 ± 3.32, which is ranging from 14 to 26. The average copy number and consensus size of tandem repeat were 2.76 ± 3.02 and 21.24 ± 14.26, respectively, while the GC content of tandem repeat was relatively low with the value of 21.98 ± 12.14.

**Figure 3:**
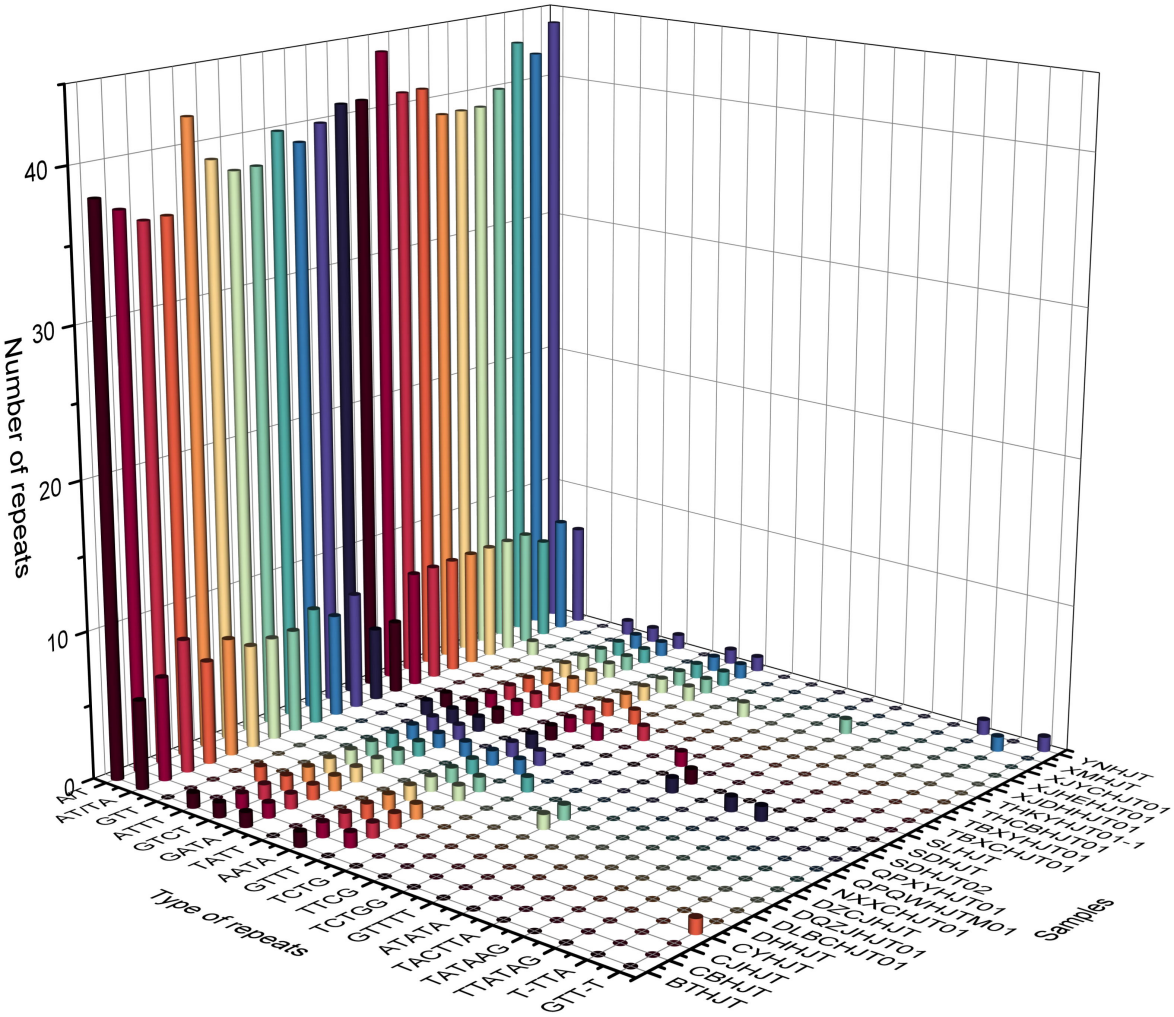
Histogram of the number and types of repeat sequences in the plastomes of *Rhodiola* genus. The X-axis represents the type of repeat sequences, the Y-axis represents the sample names, and the Z-axis represents the number of repeat sequences.

For repeat structures, the species with the fewest number of repeat structures was *R. yunnanensis* (17), while the species with the highest number of repeat structures was *R. crenulata* (45). The number of palindromic matches was significantly greater than that of direct matches, with averages of 15.74 and 9.61, respectively. Further analysis revealed 5 repeat structures shared among all species, and the 90 repeat structures were unique to a particular species (**Supplementary Table 5**). Overall, these repeat features, whether they are SSRs, tandem repeats, or repeat structures, are mainly located in intergenic spacer regions (**Figure 3**; **Supplementary Tables 6**–**8**).

### 3.4 Comparative genome analysis and IR boundaries

**Figure 4:**
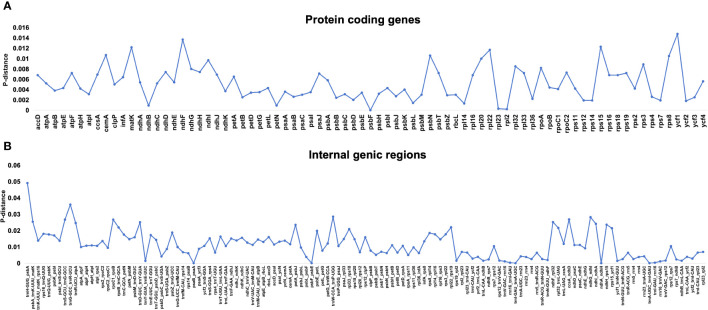
The nucleotide variability (Pi) values of 23 *Rhodiola* samples were compared. **(A)** The P‐distance values of protein coding genes. **(B)** The P‐distance values of internal genic regions.

To determine divergence and rearrangements in the plastomes of *Rhodiola*, multiple sequence alignments were conducted. The results of the chloroplast genome structure comparison using MAUVE showed that all the *Rhodiola* genome structures without gene rearrangements (**Supplementary Figure 2**). The LSC/IR and IR/SSC boundaries were conserved in JLB and JLA, but with a little variation in JSB and JSA. The length of *ndhF* gene falling into SSC is 14–61 bp, and *ycf1* falling into SSC is 4060–4108 bp, which is mainly due to changes in the 3’ end repeats of the *ndhF* and *ycf1* genes resulting in stop codon frameshifts (**Supplementary Figure 3**). For the protein coding genes, the genes with the highest sequence variation were *ycf1*, *ndhF* and *rps8*. For IGS (internal genic region) regions, *trnH-psbA, rpoC1-rpoB* and *trnW-trnP* had the highest variability. The Pi value of the most variable IGS (*trnH-psbA*) was 3.49 times than that of protein coding gene (*ycf1*) with the highest sequence variation (**Figure 4**).

### 3.5 Phylogenetic analyses of *Rhodiola* species revealed by plastomes

**Figure 5:**
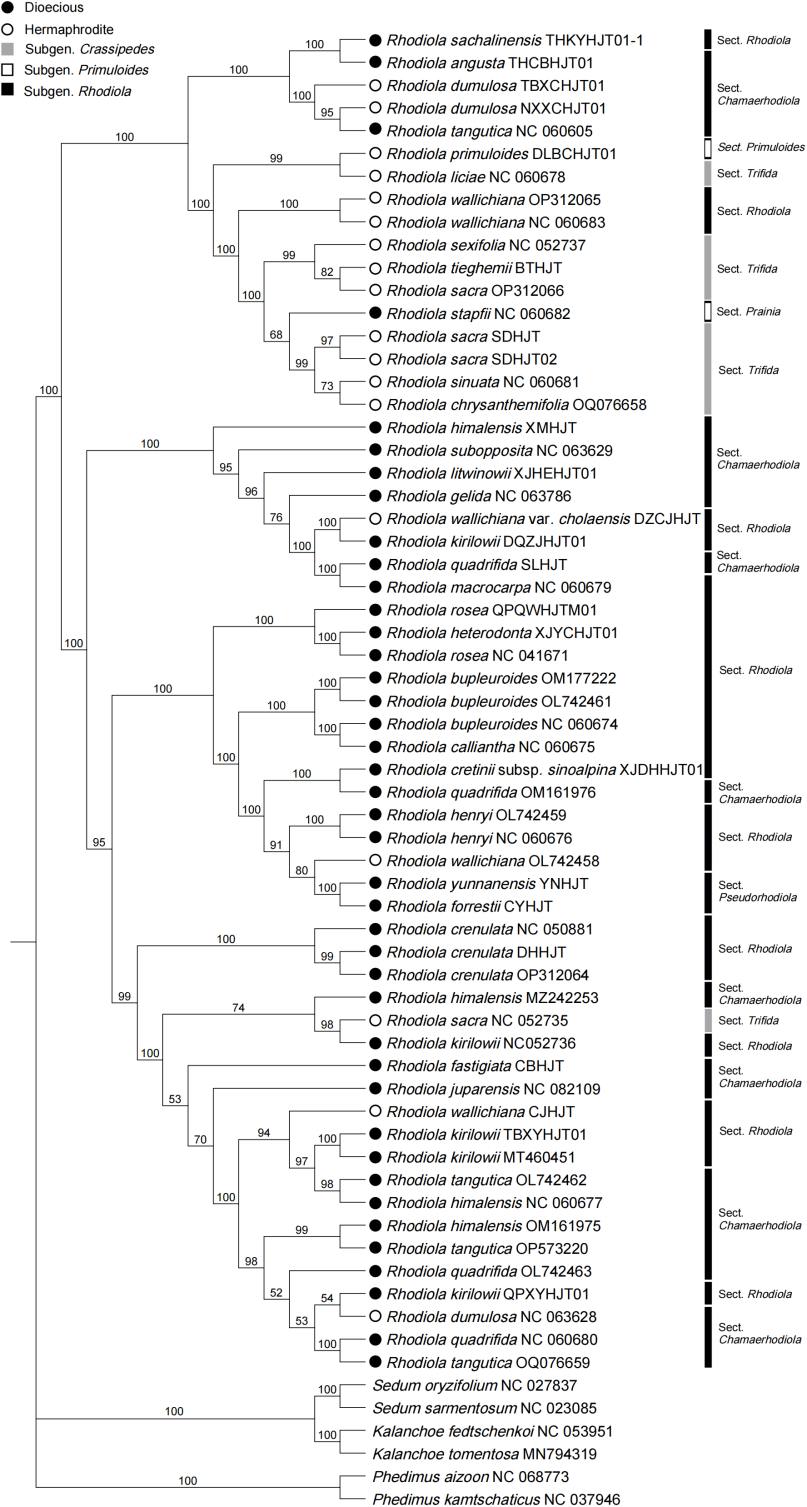
Phylogenetic tree based on CDSs shared by *Rhodiola* plastomes. Six species were selected as outgroups from other genera of the Crassulaceae.

A phylogenetic tree based on coding DNA sequences (CDSs) was constructed (**Figure 5**), and strong support was found for the clade composed of all the *Rhodiola* species, with a bootstrap value of 100. Within *Rhodiola*, none of the subgenera are monophyletic, and some subclades with greatly improved resolution compared with the ITS data (**Supplementary Figure 4**) and single plastid markers, such as *matK* (**Supplementary Figure 5**), *rbcL* (**Supplementary Figure 6**) and *psbA* (**Supplementary Figure 7**). Three representatives of *R. crenulata* formed a well-supported clade. This finding also supports the monophyly of *R. forrestii* and *R. yunnanensis*, which belong to *R.* Sect. *Pseudorhodiola. Rhodiola dumulosa* and *R. kirilowii* are polyphyletic, which is related to their rapid radiative evolution patterns. The sexual system is traditionally considered an important feature in the classification of *Rhodiola*. Dioecious species and hermaphrodite species form two main subclades, although these subclades are disrupted by a small number of other species. For example, four species (*R. angusta, R. sachalinensis, R. tangutica*, and *R. stapfii*) were dioecious but were surrounded by branches dominated by hermaphrodite species. Similarly, *R. wallichiana, R. wallichiana* var. *cholaensis*, *R. dumulosa* and *R. sacra*, which are hermaphrodites, mixed with the dioeicous subclade.

## 4 Discussion

This study sequenced and assembled the plastomes of 23 samples from 18 *Rhodiola* species, analyzing structural features, codon usage bias, repeat sequences, comparative genomics, and phylogenetic relationships. The length of *Rhodiola* plastomes ranges from 150,893 bp to 151,946 bp, and the genome size, gene order, gene number, and genome structure are highly conserved within *Rhodiola* plastomes, which is consistent with previous research (Zhao et al., 2022). In the codon analysis, we found that the average number of codons for *Rhodiola* species was greater than that for species distributed over a wide geographical range, such as *Arabidopsis thaliana.* This reflects that *Rhodiola* species have complex and diverse genomic coding requirements for adaptation to high-altitude environments. Scatter plots of ENC and GC3 detected changes in codon usage compared to the standard equations without selection pressure. Most of the genes were found not to conform to the standard equations, suggesting that in addition to nucleotide composition, other factors influence the codon usage bias of *Rhodiola* plastid genes (Guisinger et al., 2011; Gao et al., 2023). Codon usage bias generally reflects the selection pressure of optimizing the translation of highly expressed genes (Arella et al., 2021). For instance, the codon usage bias of photosystem II genes varies greatly, such as *psbF, psbJ, psbL, psbM*, and *psbT* genes, over 50% of codons have an RSCU value equal to 0, while some codons have an RSCU value of up to 6. This may reflect selective pressures on photosystem II genes for acclimation to high-altitude regions (Trošt Sedej et al., 2020). Previous studies have identified the *ndhA*, *ndhH* and *rpl16* genes as potentially crucial for adaptation of *Rhodiola* to high altitudes (Zhao et al., 2020). Our investigation revealed that while the size, number, and order of plastid genes remained largely conserved, there were discernible taxon-specific variations at the IR boundaries. Notably, *ndhF* and *ycf1* genes exhibited the obvious variation, suggesting the possibility of adaptive evolution. Repeat sequence analysis can provide candidate markers for phylogenetic and species identification. Interestingly, we found three unique repeat regions that have not been previously reported, compounded with trinucleotide repeats and monomeric repeats, which were found in *R. forrestii* (GTT-T), *R. yunnanensis* (GTT-T), and *R. himalensis* (T-TTA). These can be used as specific recognition sites for identifying *Rhodiola* species. In the infrageneric phylogenetic analysis of *Rhodiola*, we observed a polyphyletic distribution within each subgenus, indicating a complex evolutionary history. The *R.* Sect. *Rhodiola* is also polyphyletic, contains multiple species and is widely distributed. In the CDSs phylogenetic tree, three samples of *R. crenulata* formed a monophyletic group, with low genetic diversity within the species, suggesting a close association with the migration dynamics of *R. crenulata*. Moreover, *R. crenulata*, *R. himalensis*, *R. kirilowii*, and *R. tangutica* were clustered together in a subclude. However, the ITS phylogenetic tree revealed a different clustering pattern. *Rhodiola crenulata* clustered with *R. cretinii* subsp. *sinoalpina*, *R. dumulosa*, *R. angusta*, and *R. sachalinensis* in a subclude. This nuclear-cytoplasmic phylogenetic tree conflicts situation may be due to the frequent hybridization events of *Rhodiola* during rapid radiation. Similarly, cyto-nuclear discordance has also been reported in various genera such as *Cotoneaster* (Meng et al., 2021) and *Atractylodes* (Liu et al., 2022). Therefore, to further elucidate the evolutionary trajectory of *Rhodiola* species, additional haplotypes should be collected to uncover potential genetic insights. Notably, in our study, *R. forrestii* and *R. yunnanensis* converged into one branch and belonged to *R.* Sect. *Pseudorhodiola*. In previous studies, the monophyly of *R.* Sect. *Pseudorhodiola* was similarly supported, along with the single origin of the paniculate inflorescences of *Rhodiola* (Zhang et al., 2014a). Especially, both *R. forrestii* and *R. yunnanensis* have unique repeat sequences of GTT-T, prompting further investigation into whether this feature correlates with the evolution of their inflorescence traits. In CDSs tree, it can be roughly divided into two subclades based on sexual systems, although occasionally interrupted by certain species. Of particular interest is *R. wallichiana*, noted in the *Flora of China* for its hermaphroditic nature, while some specimens exhibit dioecious traits. This population may represent a transitional taxon in the evolutionary trajectory of sexual systems. Consequently, it is hypothesized that species such as *R. wallichiana* var. *cholaensis*, *R. dumulosa* and *R. sacra* may also represent transitional taxa. The evolutionary pathway of dioecious plants in *Rhodiola* is still unclear, and more samples need to be collected to explore the evolutionary patterns of trait characteristics.

The chloroplast genome can be used to detect genetic diversity within populations and reveal the geographical distribution patterns and evolutionary processes of species. *Rhodiola* serves as an ideal model for studying the adaptive evolution of plastid genes under rapid radiation. Most *Rhodiola* species are confined to high elevations on the Qinghai-Tibetan Plateau and adjacent areas, such as *R. crenulata* and *R. sacra*, which are found at altitudes ranging from 3000 to 5600 m. Zhang et al. (2018b), through the study of cpDNA and ITS datasets, found glacial refugia both on the Qinghai-Tibetan Plateau and in the Hengduan Mountains during the Last Glacial Maximum period. *Rhodiola crenulata* only grew on glacial relics, indicating its speciation during the Pleistocene epoch when suitable habitats emerged. Due to the narrow confinement of *R. crenulata* to mountain peaks and its limited population size, genetic diversity within the population remains relatively low. Milankovitch climate oscillations might repeatedly eliminate new mutations, as individuals carrying these mutations often face extinction during these oscillations. In our phylogenetic study, chloroplast genomes of *R. crenulata* collected at high altitudes in Tibet, and downloaded from NCBI, clustered into one branch despite originating from different habitats, suggesting consistently low genetic diversity within the population, consistent with previous studies. Moreover, numerous species survive in lower altitude mountain grasslands. For instance, *R. dumulosa* and *R. yunnanensis* are found in Northeast Asia at elevations ranging from 1000 to 4100 m. Hou and Lou (2014) collected 19 populations from three major distribution areas in northern, central, and northwestern China and analyzed the systematic geographic pattern of *R. dumulosa* based on four chloroplast genome fragments. The total genetic diversity of *R. dumulosa* was found to be remarkably high, likely due to long-term isolation of natural populations. They proposed a two-step migration process, suggesting that *R. dumulosa* first migrated northward from refugia on the Qinghai-Tibet Plateau *via* the intervening highlands when temperatures rose, and then migrated toward the mountaintop due to its preference for colder climates. Our data also indicate that the intraspecific genetic diversity of *R. dumulosa* is very high, possibly due to strong genetic differentiation during migration. Notably, *R. rosea* often grows at altitudes of 1800 to 2700 m and exhibits an intercontinental distribution, spanning from East Asia to the Arctic regions, Europe, and eastern North America. Gyorgy et al. (2018) sequenced six loci of the chloroplast genome of *R. rosea* from a collection of 44 sites across Eurasia, including Asia, the Carpathians, Alps, British Isles, and Scandinavia. Their results support the intercontinental migration pattern of *R. rosea* into Europe *via* the Central Asian highland corridor, reaching the European Alpine System (EAS) and the western edge of Europe, including the British Isles. The EAS is identified as a significant center of high genetic variation, particularly in the region of the Eastern Alps and the Dolomites, where glacial refugia may have existed. Exploring the rapid radiation processes of *Rhodiola* species with varying altitudinal ranges and intercontinental distributions can provide valuable insights into the evolutionary patterns of the genus *Rhodiola*.

## 5 Conclusions

Genomic analysis revealed that *Rhodiola* species exhibit a relatively conserved chloroplast genome structure, consisting of 79 protein-coding genes, 30 tRNA genes, and 4 rRNA genes. Interestingly, we observed a wide range of RSCU values for codons within the photosystem II genes, indicating selective pressure for optimizing gene translation. Furthermore, we identified unique repeat sequence structures that combine trinucleotides and monomeric repeats. These findings offer specific molecular markers for species identification. Our phylogenetic analysis strongly supported the monophyly of *R. crenulata* and *R.* Sect. *Pseudorhodiola* branches. Notably, the phylogenetic trees exhibited cyto-nuclear discordance situation. Therefore, it is essential to collect more samples to explore the evolutionary patterns within the genus. The results of our analysis provide genetic information data for the *Rhodiola* genus, providing a basis for the analysis of phylogenetic and evolutionary relationships.

## Data availability statement

The datasets presented in this study can be found in online repositories. The names of the repository/repositories and accession number(s) can be found in the article/**Supplementary Material**.

## Author contributions

JL: Conceptualization, Funding acquisition, Writing – original draft. EZ: Methodology, Writing – original draft, Writing – review & editing. YT: Formal analysis, Writing – review & editing. LZ: Investigation, Writing – review & editing. YL: Resources, Writing – review & editing. LS: Data curation, Project administration, Writing – review & editing. LX: Supervision, Writing – review & editing. PX: Funding acquisition, Writing – review & editing.
