# Supplementary material for: Comparative chloroplast genomes: insights into the identification and phylogeny of rapid radiation genus Rhodiola
Source: Front Plant Sci. 2024 May 10;15:1404447. doi: 10.3389/fpls.2024.1404447 (PMC11116683; doi:10.3389/fpls.2024.1404447)
Supplement: Supplementary file 1 [file DataSheet_1.docx]

Supplementary Material

# Supplementary Figures and Tables

## Supplementary Figures

##
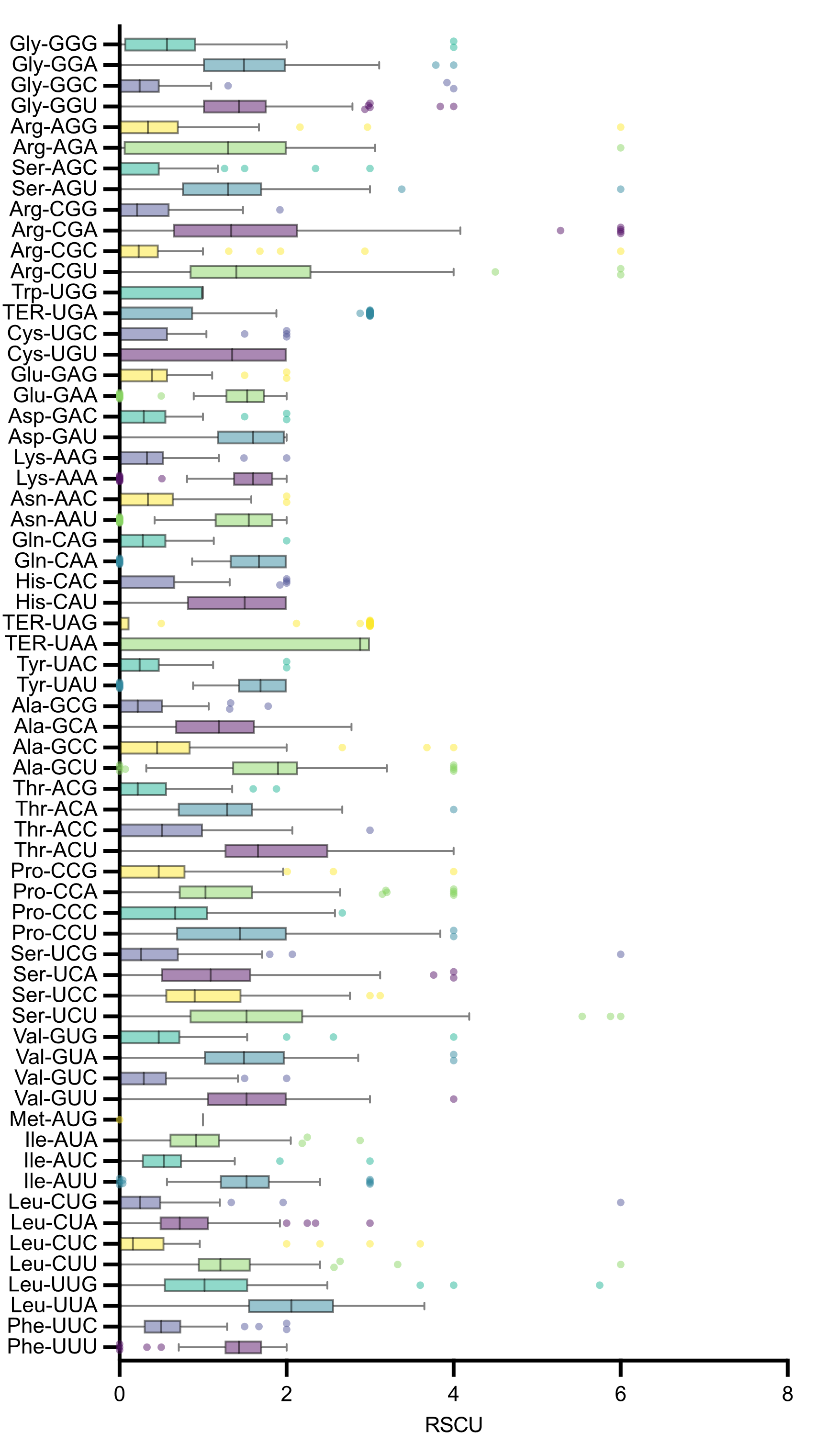


## Supplementary Figure 1. The Relative synonymous codon usage (RSCU) of the *Rhodiola* chloroplast genomes.
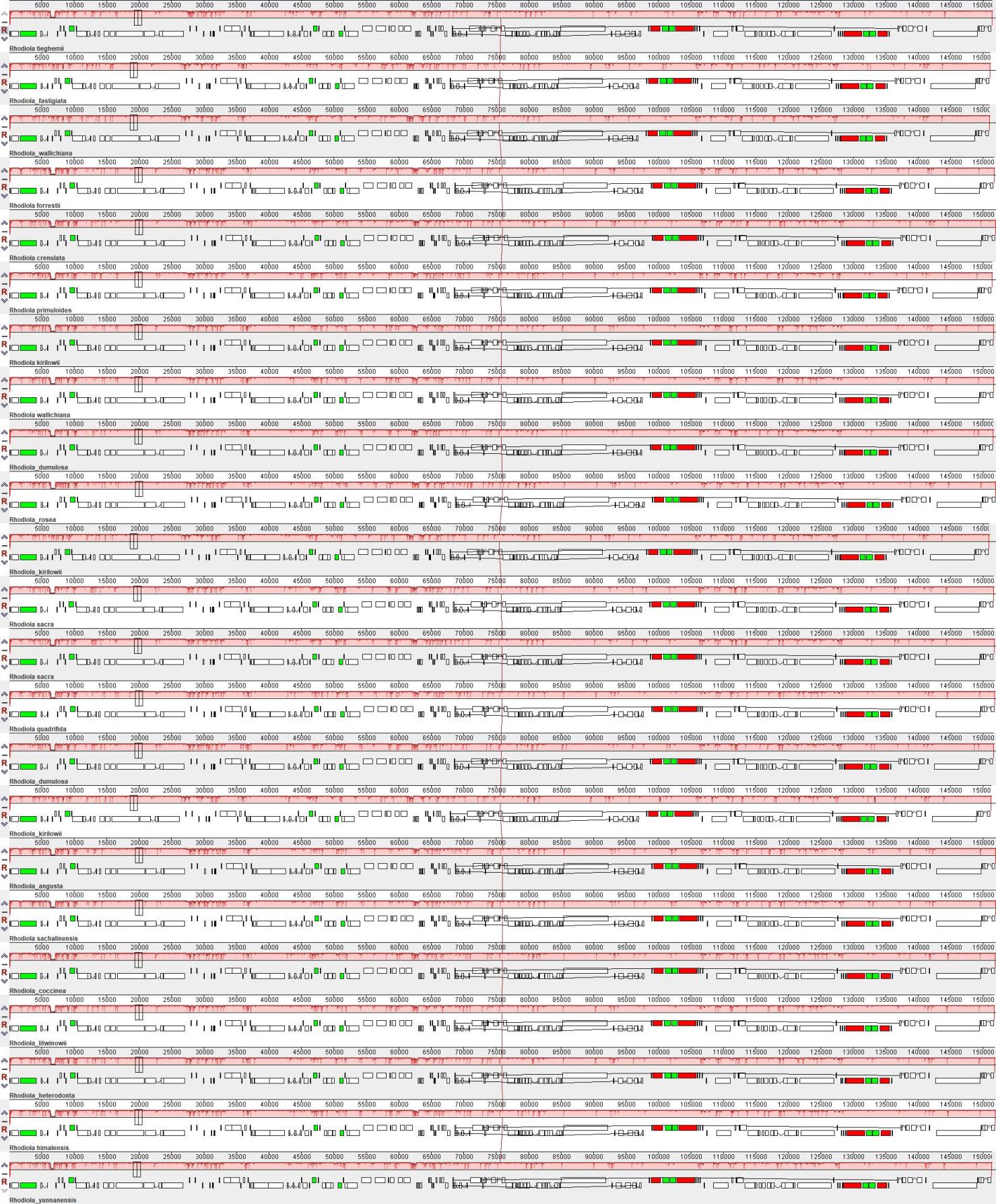


**Supplementary Figure 2.** Comparison results of *Rhodiola* chloroplast genome structures using MAUVE.


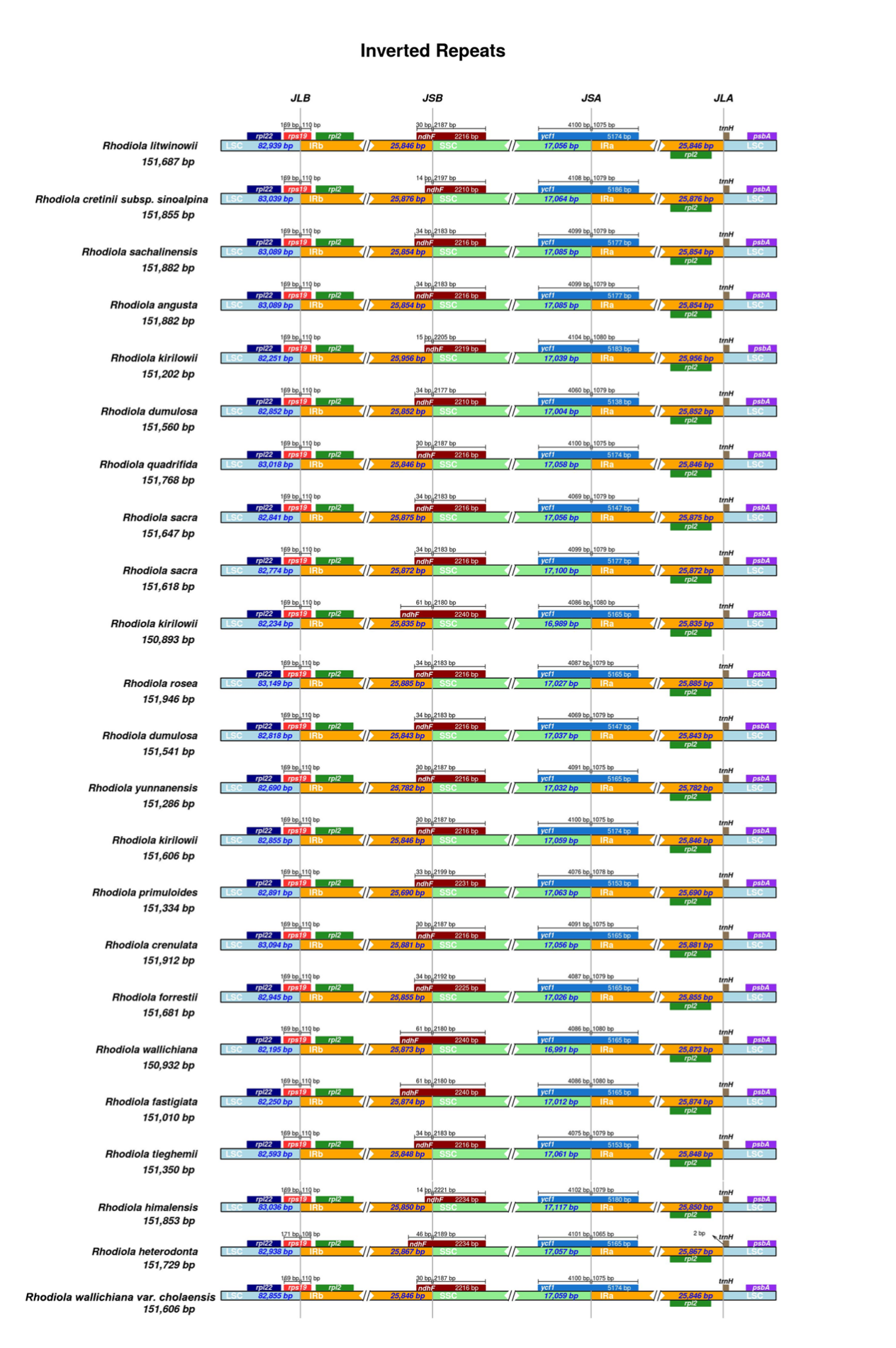


**Supplementary Figure 3.** Comparison results of *Rhodiola* chloroplast genome structures using IRScope.


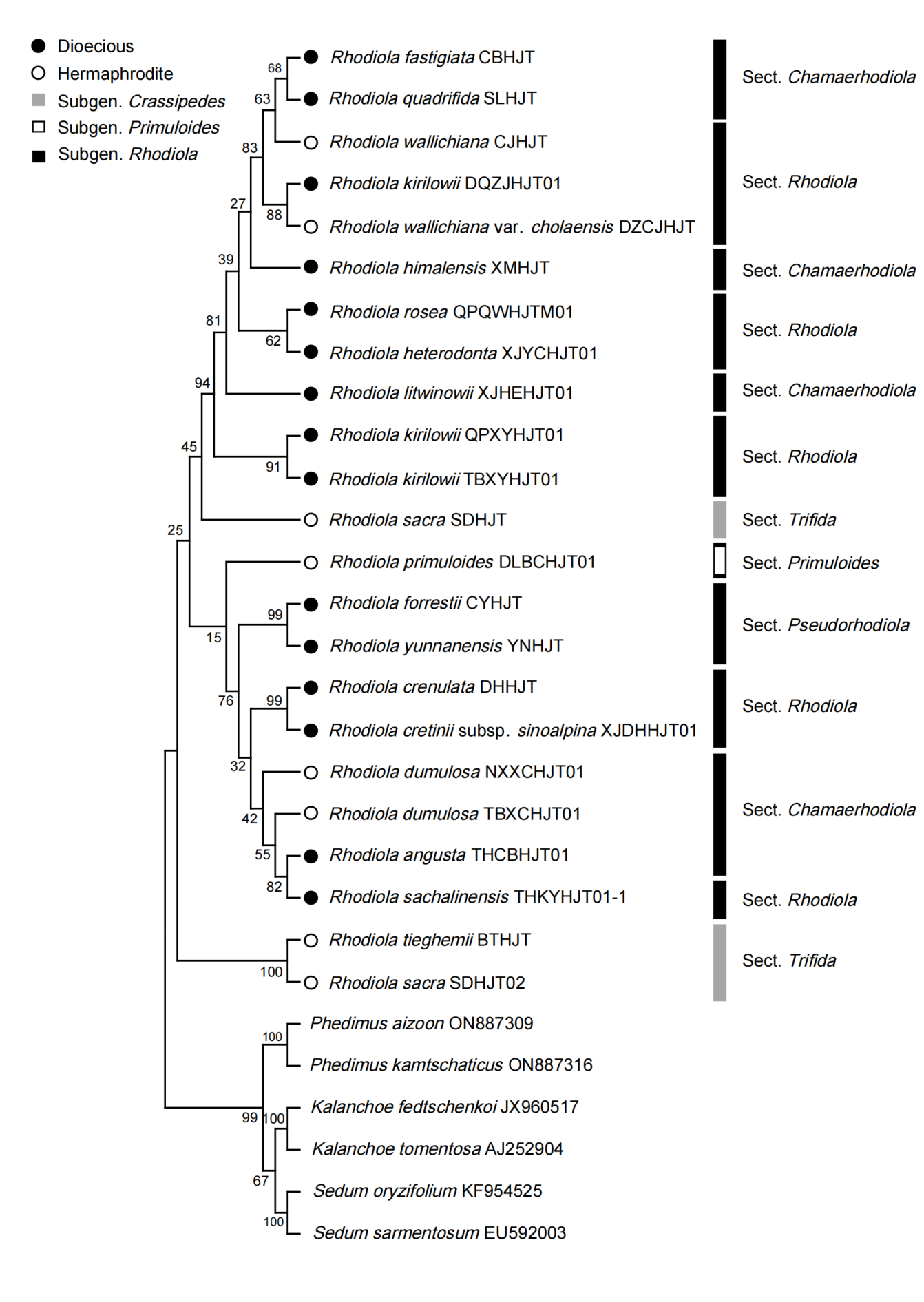


**Supplementary Figure 4.** The phylogenetic tree of *Rhodiola* based on ITS data.


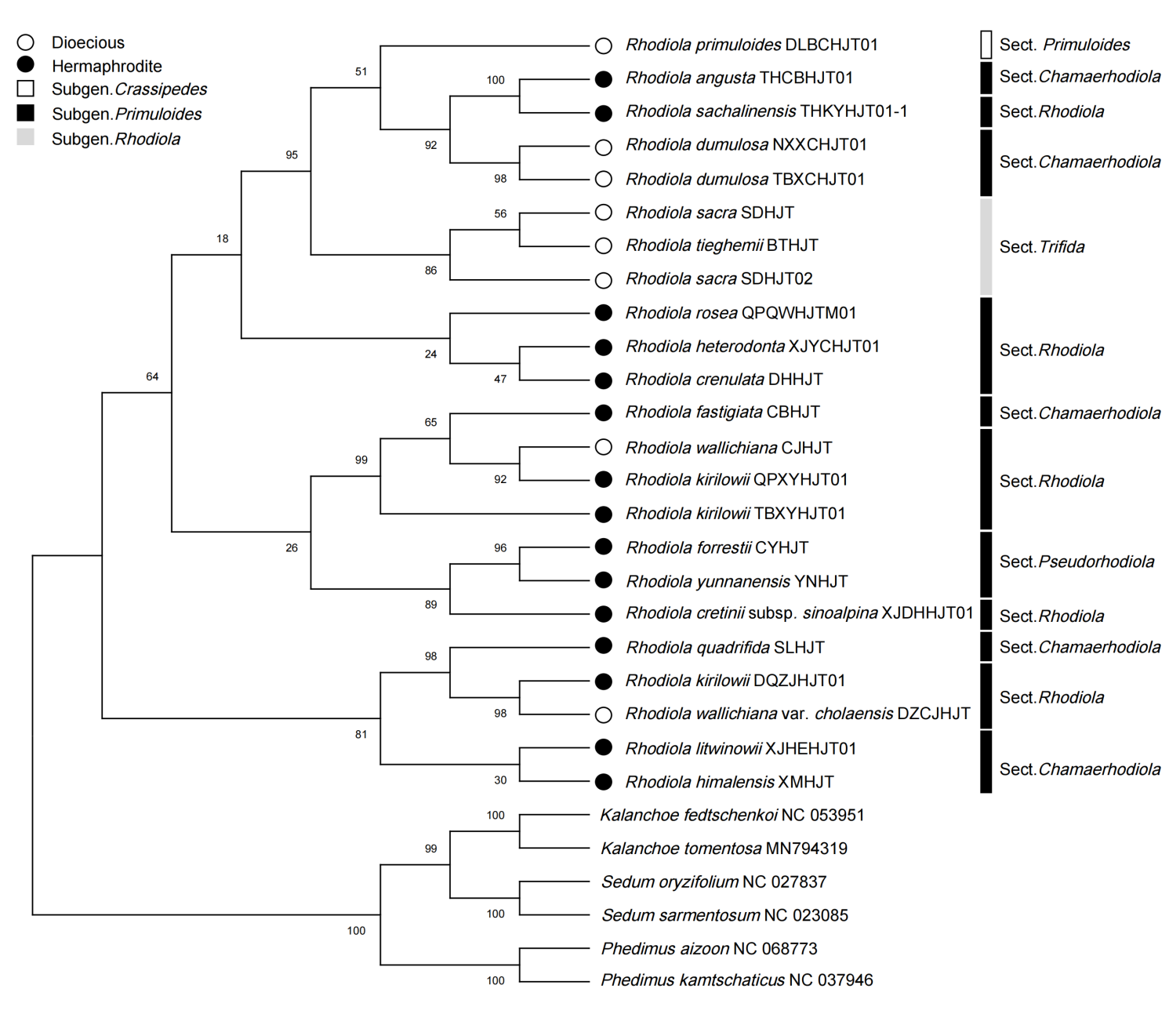


**Supplementary Figure 5.** The phylogenetic tree of *Rhodiola* based on *matK* gene.


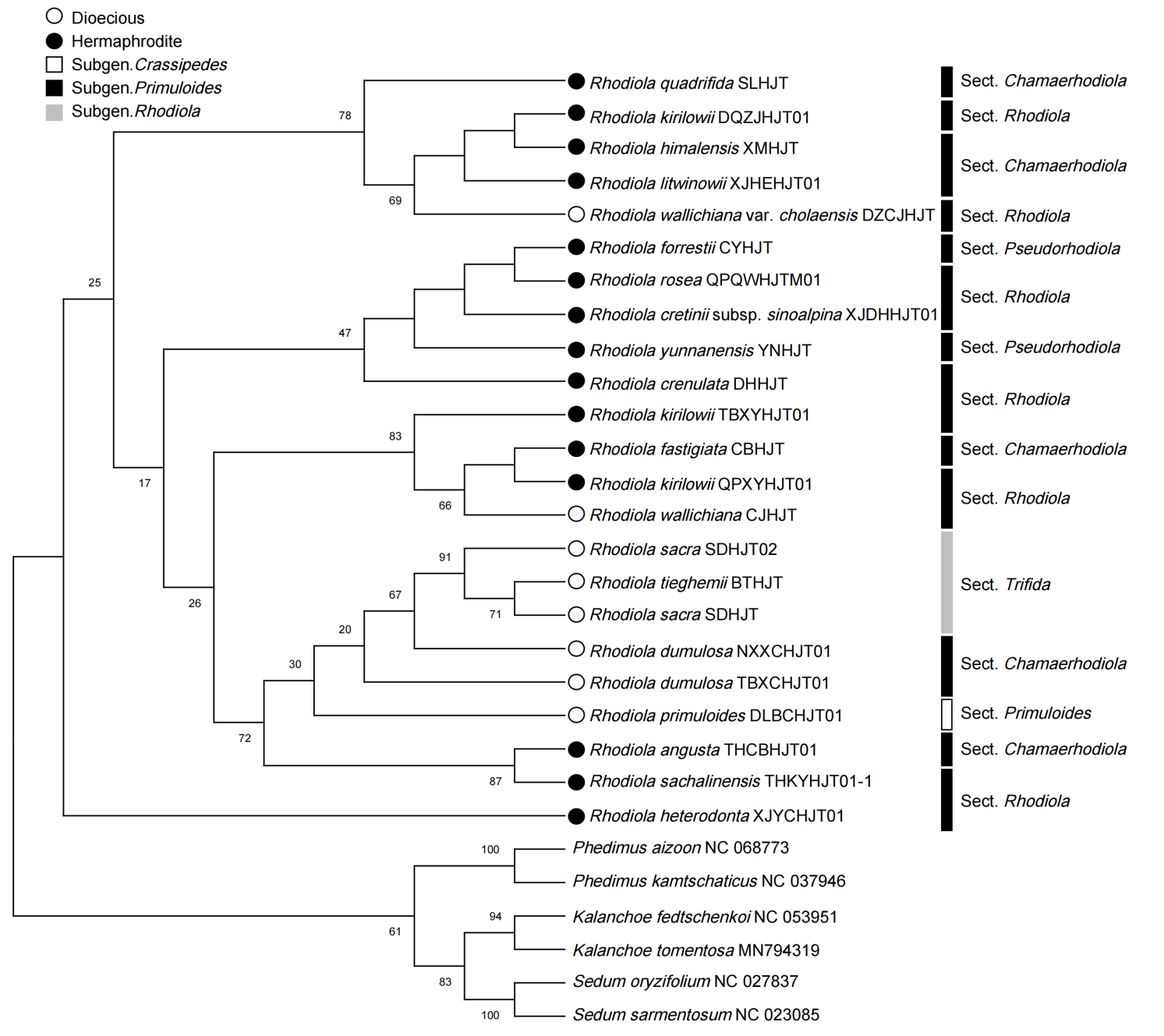


**Supplementary Figure 6.** The phylogenetic tree of *Rhodiola* based on *rbcL* gene.

**Supplementary Figure 7.** The phylogenetic tree of *Rhodiola* based on *psbA* gene.

## Supplementary Tables

**Supplementary Table 1.** The location, GenBank accession numbers, Biosample and SRA information of 23 *Rhodiola* samples.

| Sample name | Latin name | Location | GenBank accession numbers | Biosample | SRA |
| --- | --- | --- | --- | --- | --- |
| BTHJT | *Rhodiola tieghemii* | Tibet | PP262133 | SAMN39692801 | SRR27867744 |
| CBHJT | *Rhodiola fastigiata* | Tibet | PP262134 | SAMN39692787 | SRR27867738 |
| CJHJT | *Rhodiola wallichiana* | Tibet | PP262135 | SAMN39692802 | SRR27867743 |
| CYHJT | *Rhodiola forrestii* | Yunnan | PP262136 | SAMN39692788 | SRR27867737 |
| DHHJT | *Rhodiola crenulata* | Tibet | PP262137 | SAMN39692784 | SRR27867741 |
| DLBCHJT01 | *Rhodiola primuloides* | Yunnan | PP262138 | SAMN39692794 | SRR27867752 |
| DQZJHJT01 | *Rhodiola kirilowii* | Yunnan | PP262152 | SAMN39692796 | SRR27867750 |
| DZCJHJT | *Rhodiola wallichiana* var. *cholaensis* | Yunnan | PP262153 | SAMN39692782 | SRR27867756 |
| NXXCHJT01 | *Rhodiola dumulosa* | Ningxia | PP262139 | SAMN39692785 | SRR27867740 |
| QPQWHJTM01 | *Rhodiola rosea* | Hebei | PP262140 | SAMN39692797 | SRR27867749 |
| QPXYHJT01 | *Rhodiola kirilowii* | Hebei | PP262141 | SAMN39692791 | SRR27867755 |
| SDHJT | *Rhodiola sacra* | Tibet | PP262154 | SAMN39692799 | SRR27867747 |
| SDHJT02 | *Rhodiola sacra* | Yunnan | PP262142 | SAMN39692800 | SRR27867746 |
| SLHJT | *Rhodiola quadrifida* | Tibet | PP262143 | SAMN39692795 | SRR27867751 |
| TBXCHJT01 | *Rhodiola dumulosa* | Shaanxi | PP262144 | SAMN39692786 | SRR27867739 |
| TBXYHJT01 | *Rhodiola kirilowii* | Shaanxi | PP262145 | SAMN39692792 | SRR27867754 |
| THCBHJT01 | *Rhodiola angusta* | Jilin | PP262146 | SAMN39692783 | SRR27867745 |
| THKYHJT01-1 | *Rhodiola sachalinensis* | Jilin | PP262147 | SAMN39692798 | SRR27867748 |
| XJDHHJT01 | *Rhodiola cretinii* subsp. *sinoalpina* | Xinjiang | PP262148 | SAMN39692781 | SRR27867757 |
| XJHEHJT01 | *Rhodiola litwinowii* | Xinjiang | PP262149 | SAMN39692793 | SRR27867753 |
| XJYCHJT01 | *Rhodiola heterodonta* | Xinjiang | PP262150 | SAMN39692789 | SRR27867736 |
| XMHJT | *Rhodiola himalensis* | Tibet | PP262151 | SAMN39692790 | SRR27867735 |
| YNHJT | *Rhodiola yunnanensis* | Yunnan | PP262155 | SAMN39692803 | SRR27867742 |

Supplementary Table 2. Gene annotation of the *Rhodiola* chloroplast genomes.

| Category | Group | Genes |
| --- | --- | --- |
| Photosynthesis related genes | Rubisco | *rbcL* |
|  | Photosystem I | *psaA, psaB, psaC, psaI, psaJ* |
|  | Photosystem II | *psbA, psbB, psbC, psbD, psbE, psbF, psbH, psbI, psbJ, psbK, psbL, psbM, psbN, psbT, psbZ* |
|  | ATP synthase | *atpA, atpB, atpE, atpF^a^, atpH, atpI* |
|  | Cytochrome b/f complex | *petA, petB^a^, petD^a^, petN, petL, petG* |
|  | Cytochrome csynthesis | *ccsA* |
|  | Complex I of chloroplasts | *ndhA^a^, ndhB^a,c^, ndhC, ndhD, ndhE, ndhF, ndhH, ndhG, ndhJ, ndhK, ndhI* |
| Transcription and translation related genes | Transcription | *rpoA, rpoB, rpoC2, rpoC1^a^* |
|  | Ribosomal proteins | *rpl14, rpl16, rpl2^a,c^, rpl20, rpl22, rpl23^c^, rpl32, rpl33, rpl36, rps11, rps12^b,c^, rps14, rps15, rps16^a^, rps18, rps19, rps2, rps3, rps4, rps7^c^, rps8* |
|  | Translation initiation factor | *infA* |
| RNA genes | Ribosomal RNA | *rrn16S^c^, rrn23S^c^, rrn4.5^c^, rrn5^c^* |
|  | Transfer RNA | *trnA-UGC^a,c^, trnC-GCA, trnD-GUC, trnE-UUC, trnF-GAA, trnG-GCC^a^, trnG-UCC, trnH-GUG, trnI-CAU^c^, trnI-GAU^a,c^, trnK-UUU^a^, trnL-CAA^c^, trnL-UAA^a^, trnL-UAG, trnM-CAU, trnN-GUU^c^, trnP-UGG, trnQ-UUG, trnR-ACG^c^, trnR-UCU, trnS-GCU, trnS-GGA, trnS-UGA, trnT-GGU, trnT-UGU, trnV-GAC^c^, trnV-UAC^a^, trnW-CCA, trnY-GUA, trnfM-CAU* |
| Other genes | RNA processing | *matK* |
|  | Carbon metabolism | *cemA* |
|  | Fatty acid synthesis | *accD* |
|  | Proteolysis | *clpP^b^* |
|  | Conserved ORFs | *ycf1, ycf2^c^, ycf3^b^, ycf4* |

^a^genes with one intron, ^b^genes with two introns, ^c^Two gene copies in IRs.

**Supplementary Table 3.** The codon suage analysis of Rhodiola.

| Samples | T3s | C3s | A3s | G3s | CAI | CBI | Fop | Nc | GC3s | GC | L_sym | L_aa | Gravy | Aromo |
| --- | --- | --- | --- | --- | --- | --- | --- | --- | --- | --- | --- | --- | --- | --- |
| BTHJT | 0.4761 | 0.1615 | 0.4363 | 0.1706 | 0.166 | -0.102 | 0.352 | 48.9 | 0.255 | 0.377 | 21641 | 22584 | -0.041711 | 0.111008 |
| CBHJT | 0.4756 | 0.1622 | 0.4365 | 0.1701 | 0.167 | -0.1 | 0.353 | 48.92 | 0.255 | 0.377 | 21652 | 22602 | -0.042983 | 0.111273 |
| CJHJT | 0.4753 | 0.1624 | 0.4367 | 0.1701 | 0.167 | -0.1 | 0.353 | 48.96 | 0.255 | 0.377 | 21648 | 22596 | -0.042065 | 0.111391 |
| CYHJT | 0.4752 | 0.1624 | 0.4352 | 0.1717 | 0.167 | -0.1 | 0.353 | 49.02 | 0.257 | 0.378 | 21647 | 22591 | -0.042362 | 0.111195 |
| DHHJT | 0.4755 | 0.1618 | 0.4359 | 0.1713 | 0.167 | -0.101 | 0.352 | 48.96 | 0.256 | 0.377 | 21649 | 22596 | -0.04233 | 0.111303 |
| DLBCHJT01 | 0.4755 | 0.1622 | 0.436 | 0.1707 | 0.166 | -0.101 | 0.352 | 48.96 | 0.256 | 0.377 | 21647 | 22594 | -0.04233 | 0.111224 |
| DQZJHJT01 | 0.475 | 0.1621 | 0.4368 | 0.1708 | 0.166 | -0.101 | 0.352 | 48.94 | 0.256 | 0.377 | 21658 | 22607 | -0.042248 | 0.111293 |
| DZCJHJT | 0.475 | 0.1621 | 0.4368 | 0.1708 | 0.166 | -0.101 | 0.352 | 48.94 | 0.256 | 0.377 | 21658 | 22607 | -0.042279 | 0.111293 |
| NXXCHJT01 | 0.4753 | 0.1623 | 0.4354 | 0.1712 | 0.167 | -0.101 | 0.352 | 49.01 | 0.256 | 0.378 | 21631 | 22574 | -0.040844 | 0.11119 |
| QPQWHJTM01 | 0.4755 | 0.1619 | 0.4368 | 0.1704 | 0.167 | -0.101 | 0.352 | 48.92 | 0.255 | 0.377 | 21652 | 22604 | -0.041533 | 0.111308 |
| QPXYHJT01 | 0.4753 | 0.1624 | 0.4366 | 0.1701 | 0.167 | -0.1 | 0.353 | 48.96 | 0.255 | 0.377 | 21656 | 22605 | -0.040659 | 0.111436 |
| SDHJT02 | 0.4761 | 0.1618 | 0.4358 | 0.1705 | 0.167 | -0.101 | 0.352 | 48.94 | 0.255 | 0.377 | 21647 | 22591 | -0.041309 | 0.11115 |
| SDHJT | 0.4759 | 0.162 | 0.4356 | 0.1708 | 0.167 | -0.101 | 0.352 | 48.96 | 0.256 | 0.377 | 21633 | 22578 | -0.041775 | 0.11117 |
| SLHJT | 0.4749 | 0.1622 | 0.4366 | 0.171 | 0.166 | -0.101 | 0.352 | 48.96 | 0.256 | 0.377 | 21658 | 22606 | -0.043303 | 0.111077 |
| TBXCHJT01 | 0.4757 | 0.162 | 0.4355 | 0.1712 | 0.167 | -0.101 | 0.352 | 49 | 0.256 | 0.377 | 21625 | 22569 | -0.03896 | 0.111215 |
| TBXYHJT01 | 0.4755 | 0.1623 | 0.4366 | 0.1703 | 0.167 | -0.101 | 0.353 | 48.98 | 0.255 | 0.377 | 21646 | 22595 | -0.041916 | 0.111441 |
| THCBHJT01 | 0.4752 | 0.1624 | 0.4356 | 0.1712 | 0.167 | -0.101 | 0.352 | 48.98 | 0.256 | 0.377 | 21639 | 22587 | -0.042086 | 0.110993 |
| THKYHJT01-1 | 0.4752 | 0.1624 | 0.4356 | 0.1712 | 0.167 | -0.101 | 0.352 | 48.98 | 0.256 | 0.377 | 21639 | 22587 | -0.042086 | 0.110993 |
| XJDHHJT01 | 0.475 | 0.1622 | 0.4364 | 0.1708 | 0.166 | -0.1 | 0.353 | 48.97 | 0.256 | 0.377 | 21644 | 22588 | -0.041513 | 0.1109 |
| XJHEHJT01 | 0.4753 | 0.162 | 0.4362 | 0.1712 | 0.166 | -0.102 | 0.352 | 48.95 | 0.256 | 0.377 | 21655 | 22602 | -0.04335 | 0.111096 |
| XJYCHJT01 | 0.4757 | 0.1618 | 0.4365 | 0.1707 | 0.167 | -0.101 | 0.353 | 48.94 | 0.255 | 0.377 | 21666 | 22610 | -0.040761 | 0.111411 |
| XMHJT | 0.4761 | 0.1617 | 0.4363 | 0.171 | 0.166 | -0.102 | 0.352 | 48.91 | 0.255 | 0.377 | 21659 | 22605 | -0.043013 | 0.111259 |
| YNHJT | 0.4748 | 0.1624 | 0.4358 | 0.1713 | 0.166 | -0.1 | 0.353 | 49 | 0.256 | 0.377 | 21635 | 22583 | -0.04061 | 0.111146 |

**Supplementary Table 4.** The number and types of SSR of *Rhodiola*.

| Samples | total | mono | | di | tri | tetra | penta | hex | repeat region | SSR type |
| --- | --- | --- | --- | --- | --- | --- | --- | --- | --- | --- |
| BTHJT | 48 | | 38 | 6 | 0 | 4 | 0 | 0 | 0 | ['T', 'A', 'AT', 'TA', 'ATTT', 'GTCT', 'GATA', 'AATA'] |
| CBHJT | 48 | | 37 | 7 | 0 | 4 | 0 | 0 | 0 | ['T', 'A', 'TA', 'AT', 'GTTT', 'GTCT', 'GATA', 'AATA'] |
| CJHJT | 49 | | 36 | 9 | 0 | 4 | 0 | 0 | 0 | ['T', 'A', 'TA', 'AT', 'GTTT', 'GTCT', 'GATA', 'AATA'] |
| CYHJT | 49 | | 36 | 7 | 0 | 5 | 0 | 0 | 1 | ['T', 'A', 'TA', 'AT', 'ATTT', 'GTTT', 'GTCT', 'GATA', 'AATA', 'GTT-T'] |
| DHHJT | 54 | | 42 | 8 | 0 | 4 | 0 | 0 | 0 | ['T', 'A', 'TA', 'AT', 'GTTT', 'GTCT', 'GATA', 'AATA'] |
| DLBCHJT01 | 49 | | 39 | 7 | 0 | 3 | 0 | 0 | 0 | ['T', 'A', 'AT', 'TA', 'GTCT', 'GATA', 'AATA'] |
| DQZJHJT01 | 50 | | 38 | 7 | 0 | 4 | 1 | 0 | 0 | ['T', 'A', 'TA', 'AT', 'GTTT', 'GTCT', 'GATA', 'AATA', 'TCTGG'] |
| DZCJHJT | 50 | | 38 | 7 | 0 | 4 | 1 | 0 | 0 | ['T', 'A', 'TA', 'AT', 'GTTT', 'GTCT', 'GATA', 'AATA', 'TCTGG'] |
| NXXCHJT01 | 52 | | 40 | 8 | 0 | 4 | 0 | 0 | 0 | ['T', 'A', 'AT', 'TA', 'GTCT', 'TCTG', 'GATA', 'AATA'] |
| QPQWHJTM01 | 51 | | 39 | 7 | 0 | 5 | 0 | 0 | 0 | ['T', 'A', 'TA', 'AT', 'GTTT', 'GTCT', 'TATT', 'GATA', 'AATA'] |
| QPXYHJT01 | 52 | | 40 | 8 | 0 | 4 | 0 | 0 | 0 | ['T', 'A', 'TA', 'AT', 'GTTT', 'GTCT', 'GATA', 'AATA'] |
| SDHJT02 | 53 | | 41 | 5 | 0 | 4 | 1 | 2 | 0 | ['T', 'A', 'AT', 'TA', 'ATTT', 'GTCT', 'GATA', 'AATA', 'GTTTT', 'TACTTA', 'TATAAG'] |
| SDHJT | 51 | | 41 | 5 | 0 | 4 | 1 | 0 | 0 | ['T', 'A', 'AT', 'TA', 'ATTT', 'GTCT', 'GATA', 'AATA', 'GTTTT'] |
| SLHJT | 57 | | 44 | 8 | 0 | 4 | 1 | 0 | 0 | ['T', 'A', 'TA', 'AT', 'GTTT', 'GTCT', 'GATA', 'AATA', 'TCTGG'] |
| TBXCHJT01 | 53 | | 41 | 8 | 0 | 4 | 0 | 0 | 0 | ['T', 'A', 'AT', 'TA', 'GTCT', 'TCTG', 'GATA', 'AATA'] |
| TBXYHJT01 | 53 | | 41 | 8 | 0 | 4 | 0 | 0 | 0 | ['T', 'A', 'TA', 'AT', 'GTTT', 'GTCT', 'GATA', 'AATA'] |
| THCBHJT01 | 50 | | 39 | 8 | 0 | 3 | 0 | 0 | 0 | ['T', 'A', 'AT', 'TA', 'GTCT', 'GATA', 'AATA'] |
| THKYHJT01-1 | 50 | | 39 | 8 | 0 | 3 | 0 | 0 | 0 | ['T', 'A', 'AT', 'TA', 'GTCT', 'GATA', 'AATA'] |
| XJDHHJT01 | 53 | | 39 | 8 | 1 | 5 | 0 | 0 | 0 | ['T', 'A', 'TA', 'AT', 'GTT', 'GTTT', 'GTCT', 'TTCG', 'GATA', 'AATA'] |
| XJHEHJT01 | 53 | | 40 | 8 | 0 | 4 | 1 | 0 | 0 | ['T', 'A', 'TA', 'AT', 'GTTT', 'GTCT', 'GATA', 'AATA', 'ATATA'] |
| XJYCHJT01 | 54 | | 43 | 7 | 0 | 4 | 0 | 0 | 0 | ['T', 'A', 'TA', 'AT', 'GTTT', 'GTCT', 'GATA', 'AATA'] |
| XMHJT | 55 | | 42 | 8 | 0 | 4 | 0 | 0 | 1 | ['T', 'A', 'TA', 'AT', 'GTTT', 'GTCT', 'GATA', 'AATA', 'T-TTA'] |
| YNHJT | 58 | | 44 | 7 | 0 | 5 | 0 | 1 | 1 | ['T', 'A', 'TA', 'AT', 'ATTT', 'GTTT', 'GTCT', 'GATA', 'AATA', 'GTT-T', 'TTATAG'] |

**Supplementary Table 5.** The number and types of repeat structures of *Rhodiola*.

| Number | Samples | Repeat structures |
| --- | --- | --- |
| 23 | BTHJT;CBHJT;CJHJT;CYHJT;DHHJT;DLBCHJT01;DQZJHJT01;DZCJHJT;NXXCHJT01;QPQWHJTM01;QPXYHJT01;SDHJT02;SDHJT;SLHJT;TBXCHJT01;TBXYHJT01;THCBHJT01;THKYHJT01-1;XJDHHJT01;XJHEHJT01;XJYCHJT01;XMHJT;YNHJT | AACGGAAAGAGAGGGATTCGAACCCTCGGTA |
| 23 | BTHJT;CBHJT;CJHJT;CYHJT;DHHJT;DLBCHJT01;DQZJHJT01;DZCJHJT;NXXCHJT01;QPQWHJTM01;QPXYHJT01;SDHJT02;SDHJT;SLHJT;TBXCHJT01;TBXYHJT01;THCBHJT01;THKYHJT01-1;XJDHHJT01;XJHEHJT01;XJYCHJT01;XMHJT;YNHJT | AAAGGAGAGAGAGGGATTCGAACCCTCGATA |
| 23 | BTHJT;CBHJT;CJHJT;CYHJT;DHHJT;DLBCHJT01;DQZJHJT01;DZCJHJT;NXXCHJT01;QPQWHJTM01;QPXYHJT01;SDHJT02;SDHJT;SLHJT;TBXCHJT01;TBXYHJT01;THCBHJT01;THKYHJT01-1;XJDHHJT01;XJHEHJT01;XJYCHJT01;XMHJT;YNHJT | TACAGAACCATACATGAGATTTTCACCTCATACGGCTCCTC |
| 23 | BTHJT;CBHJT;CJHJT;CYHJT;DHHJT;DLBCHJT01;DQZJHJT01;DZCJHJT;NXXCHJT01;QPQWHJTM01;QPXYHJT01;SDHJT02;SDHJT;SLHJT;TBXCHJT01;TBXYHJT01;THCBHJT01;THKYHJT01-1;XJDHHJT01;XJHEHJT01;XJYCHJT01;XMHJT;YNHJT | ACGGAAAGAGAGGGATTCGAACCCTCGGTA |
| 23 | BTHJT;CBHJT;CJHJT;CYHJT;DHHJT;DLBCHJT01;DQZJHJT01;DZCJHJT;NXXCHJT01;QPQWHJTM01;QPXYHJT01;SDHJT02;SDHJT;SLHJT;TBXCHJT01;TBXYHJT01;THCBHJT01;THKYHJT01-1;XJDHHJT01;XJHEHJT01;XJYCHJT01;XMHJT;YNHJT | AAGGAGAGAGAGGGATTCGAACCCTCGATA |
| 22 | BTHJT;CBHJT;CJHJT;CYHJT;DHHJT;DLBCHJT01;DQZJHJT01;DZCJHJT;NXXCHJT01;QPQWHJTM01;QPXYHJT01;SDHJT02;SDHJT;SLHJT;TBXCHJT01;TBXYHJT01;THCBHJT01;THKYHJT01-1;XJDHHJT01;XJHEHJT01;XMHJT;YNHJT | TAGAGTAGAGAAACTAGATATATCTAGTTTCTCTACTCTA |
| 22 | BTHJT;CBHJT;CJHJT;CYHJT;DHHJT;DLBCHJT01;DQZJHJT01;DZCJHJT;NXXCHJT01;QPXYHJT01;SDHJT02;SDHJT;SLHJT;TBXCHJT01;TBXYHJT01;THCBHJT01;THKYHJT01-1;XJDHHJT01;XJHEHJT01;XJYCHJT01;XMHJT;YNHJT | ATTTATTTACTTTTCTATATTAAATAGAAAAGTAAATAAAT |
| 21 | BTHJT;CBHJT;CJHJT;CYHJT;DLBCHJT01;DQZJHJT01;DZCJHJT;NXXCHJT01;QPQWHJTM01;QPXYHJT01;SDHJT02;SDHJT;SLHJT;TBXCHJT01;TBXYHJT01;THCBHJT01;THKYHJT01-1;XJDHHJT01;XJHEHJT01;XJYCHJT01;XMHJT | CTTCATGCATAAGGCTACTAGATTACCTAGGAGAAAAGATGTTAAAATCATCACAAACCTCC |
| 21 | BTHJT;CBHJT;CJHJT;CYHJT;DHHJT;DLBCHJT01;DQZJHJT01;DZCJHJT;NXXCHJT01;QPXYHJT01;SDHJT02;SDHJT;SLHJT;TBXCHJT01;TBXYHJT01;THCBHJT01;THKYHJT01-1;XJDHHJT01;XJHEHJT01;XMHJT;YNHJT | TACAGAACCGTACATGAGATTTTCACCTCATACGGCTCCTC |
| 21 | BTHJT;CBHJT;CJHJT;CYHJT;DHHJT;DLBCHJT01;DQZJHJT01;DZCJHJT;NXXCHJT01;QPXYHJT01;SDHJT02;SDHJT;SLHJT;TBXCHJT01;TBXYHJT01;THCBHJT01;THKYHJT01-1;XJDHHJT01;XJHEHJT01;XMHJT;YNHJT | TACATGAGATTTTCACCTCATACGGCTCCTC |
| 21 | CBHJT;CJHJT;CYHJT;DHHJT;DLBCHJT01;DQZJHJT01;DZCJHJT;NXXCHJT01;QPQWHJTM01;QPXYHJT01;SDHJT02;SDHJT;SLHJT;TBXCHJT01;TBXYHJT01;THCBHJT01;THKYHJT01-1;XJHEHJT01;XJYCHJT01;XMHJT;YNHJT | TATGAAAGCATGTAAGAACTCAAGGGCACCTTCCCCCTC |
| 18 | BTHJT;CBHJT;CJHJT;CYHJT;DHHJT;DLBCHJT01;NXXCHJT01;QPQWHJTM01;QPXYHJT01;SDHJT02;SDHJT;TBXCHJT01;TBXYHJT01;THCBHJT01;THKYHJT01-1;XJDHHJT01;XJYCHJT01;YNHJT | AAAGATCTTGTATATCTAGATACTAGATATACAAGATCTTT |
| 15 | CBHJT;CJHJT;CYHJT;DHHJT;DQZJHJT01;DZCJHJT;QPQWHJTM01;QPXYHJT01;SLHJT;TBXYHJT01;XJDHHJT01;XJHEHJT01;XJYCHJT01;XMHJT;YNHJT | TGCAATAGCTAAATGATGATGAGCCATATCAGTCAGCCATA |
| 15 | CBHJT;CJHJT;CYHJT;DHHJT;DQZJHJT01;DZCJHJT;QPQWHJTM01;QPXYHJT01;SLHJT;TBXYHJT01;XJDHHJT01;XJHEHJT01;XJYCHJT01;XMHJT;YNHJT | TGCAATAGCTAAATGATGGTGTGCAATATCAGTCAGCCATA |
| 14 | CBHJT;CJHJT;CYHJT;DHHJT;DQZJHJT01;DZCJHJT;QPQWHJTM01;SLHJT;TBXYHJT01;XJDHHJT01;XJHEHJT01;XJYCHJT01;XMHJT;YNHJT | AGAAAAAGAACTGCAATAGCTAAATGATGATGAGC |
| 14 | CBHJT;CJHJT;CYHJT;DHHJT;DQZJHJT01;DZCJHJT;QPQWHJTM01;SLHJT;TBXYHJT01;XJDHHJT01;XJHEHJT01;XJYCHJT01;XMHJT;YNHJT | AGAAAAAGAATTGCAATAGCTAAATGATGGTGTGC |
| 14 | CBHJT;CJHJT;CYHJT;DHHJT;DQZJHJT01;DZCJHJT;QPQWHJTM01;QPXYHJT01;SLHJT;TBXYHJT01;XJDHHJT01;XJHEHJT01;XJYCHJT01;XMHJT | AGAAAAGATGTTAAAATCATCACAAACCTCC |
| 14 | CBHJT;CJHJT;CYHJT;DHHJT;DQZJHJT01;DZCJHJT;QPQWHJTM01;QPXYHJT01;SLHJT;TBXYHJT01;XJDHHJT01;XJHEHJT01;XJYCHJT01;XMHJT | CTAGGTAATCTAGTAGCCTTATGCATGAAG |
| 14 | CBHJT;CJHJT;CYHJT;DHHJT;DQZJHJT01;DZCJHJT;QPQWHJTM01;QPXYHJT01;SLHJT;TBXYHJT01;XJDHHJT01;XJHEHJT01;XJYCHJT01;XMHJT | CTTCATGCATAAGGCTACTAGATTACCTAGTAGAAAAGATGTTAAAATCATCACAAACCTCC |
| 12 | CBHJT;CJHJT;CYHJT;DHHJT;DQZJHJT01;DZCJHJT;QPXYHJT01;SLHJT;TBXYHJT01;XJDHHJT01;XJHEHJT01;XMHJT | AATTTAGATAATAATATTATTATCTAAGTT |
| 9 | BTHJT;DLBCHJT01;NXXCHJT01;QPXYHJT01;SDHJT02;SDHJT;TBXCHJT01;THCBHJT01;THKYHJT01-1 | AGAAAAAGAATTGCAATAGCTAAATGATGGTG |
| 8 | BTHJT;DLBCHJT01;NXXCHJT01;SDHJT02;SDHJT;TBXCHJT01;THCBHJT01;THKYHJT01-1 | AGAAAAAGAACTGCAATAGCTAAATGATGATG |
| 8 | BTHJT;DLBCHJT01;NXXCHJT01;SDHJT02;SDHJT;TBXCHJT01;THCBHJT01;THKYHJT01-1 | CTTCATGCATAAGGCTACTAGATTACCTAGGAGAAAAGGTGTTAAAATCATCACAAACCTCC |
| 8 | BTHJT;DLBCHJT01;NXXCHJT01;SDHJT02;SDHJT;TBXCHJT01;THCBHJT01;THKYHJT01-1 | CTTTTCTCCTAGGTAATCTAGTAGCCTTATGCATGAAG |
| 8 | BTHJT;DLBCHJT01;NXXCHJT01;SDHJT02;SDHJT;TBXCHJT01;THCBHJT01;THKYHJT01-1 | AACAACACGGATTACTAGTAATCCATGTTGTT |
| 8 | CBHJT;CJHJT;DQZJHJT01;DZCJHJT;QPXYHJT01;SLHJT;TBXYHJT01;XJHEHJT01 | TAGATATTAGATAATTTAGATAATAATATTA |
| 8 | CBHJT;CJHJT;DQZJHJT01;DZCJHJT;QPXYHJT01;SLHJT;TBXYHJT01;XJHEHJT01 | TAATATTATTATCTAAGTTATCTAATAATTA |
| 5 | DQZJHJT01;DZCJHJT;SLHJT;XJHEHJT01;XMHJT | AAGATCTTGTATATCTAGATACTAGATATACAAGATCTT |
| 2 | CYHJT;XMHJT | TAATTAGATAATTTAGATAATAATATTAAT |
| 2 | CYHJT;XMHJT | AATAATATTATTATCTAAGTTATCTAATAA |
| 2 | QPQWHJTM01;YNHJT | ATTAGATAATTTAGATAATAATATTATTATCTAAGTTATCTAAT |
| 2 | SDHJT;XJHEHJT01 | AAACTTCATTTTTTATTTCAATAGAAATAAAAAATGAAGTTT |
| 2 | THCBHJT01;THKYHJT01-1 | AAATCGAGGTCTTCCTCTGTATTGCTATCG |
| 2 | THCBHJT01;THKYHJT01-1 | CTATCGAGGTCTTCCTCTGTATTGCTTTCG |
| 2 | THCBHJT01;THKYHJT01-1 | ATTTTTAACTTATTCTATTTTCTATCTATTTTTAACTTATTCTATTTTCTATCTA |
| 2 | THCBHJT01;THKYHJT01-1 | ATTTTTAACTTATTCTATTTTCTATCTATT |
| 2 | THCBHJT01;THKYHJT01-1 | ATTTTTAACTTATTCTATTTTCTATCTAAT |
| 2 | THCBHJT01;THKYHJT01-1 | ATTGCATTGATATCAATAAACCTTTTCTTT |
| 2 | THCBHJT01;THKYHJT01-1 | TTTTCTTTGATATCAATAAACCTTTTCTTT |
| 2 | XJDHHJT01;YNHJT | TATATATTTCTATATAGAAATTTCTATATA |
| 2 | XJDHHJT01;YNHJT | TATATAGAAATTTCTATATATATATATATA |
| 1 | BTHJT | ATATATAAACTCAAGGGCACCTTCCCCCTC |
| 1 | BTHJT | GAGGGGGAAGGTGCCCTTGAGTTCTTACAT |
| 1 | BTHJT | AAACTTTATTTTTTATTTCAATAGAAATAAAAAAT |
| 1 | BTHJT | ATTTTTTATTTCAATAGAAATAAAAAATGAAGTTT |
| 1 | CYHJT | TGTTGAATAGTGATTTGATTCAACACCTGTTGAATA |
| 1 | CYHJT | TGTTGAATAGTGATTTGATTCAACACCTAATGTATA |
| 1 | DHHJT | AGAAAAGATATTAAAATCATCACAAACCTCC |
| 1 | DHHJT | ATATATATAGATTTATTAATATATATATAGAT |
| 1 | DHHJT | ATATATATAGATTTATATATATATATATATAT |
| 1 | DHHJT | ATTAATATATATATAGATTTATATATATAT |
| 1 | DHHJT | ATTTATATATATATATATATATATATATAT |
| 1 | DHHJT | ATCTTTTCTCCTAGGTAATCTAGTAGCCTTATGCATGAAG |
| 1 | DHHJT | ATCTTTTCTACTAGGTAATCTAGTAGCCTTATGCATGAAG |
| 1 | DHHJT | CTTCATGCATAAGGCTACTAGATTACCTAGGAGAAAAGATATTAAAATCATCACAAACCTCC |
| 1 | DHHJT | TTTATATATATATATATATATATATATATTA |
| 1 | DHHJT | TATATATATATATATATATATATATATTATA |
| 1 | DHHJT | ATAGATTTATATATATATATATATATATAT |
| 1 | DHHJT | ATATAGATTTATATATATATATATATATAT |
| 1 | DHHJT | ATATATAGATTTATATATATATATATATAT |
| 1 | DHHJT | ATATATATAGATTTATATATATATATATAT |
| 1 | DHHJT | ATATATATATAGATTTATATATATATATAT |
| 1 | DHHJT | TTTTTATTAATCATATTAATTCTTTTTATTAAT |
| 1 | DHHJT | TTTTTATTAATCATATTAATTCTATTTAATTAT |
| 1 | DHHJT | TAATATATATATAGATTTATATATATATATAT |
| 1 | DHHJT | ATTTATATATATATATATATATATATATATTA |
| 1 | DHHJT | TTATATATATATATATATATATATATATTA |
| 1 | DHHJT | ATATATATATATATATATATATATTATAAT |
| 1 | DHHJT | AGATTTATATATATATATATATATATATAT |
| 1 | DHHJT | AGAAATTTCTATATATATATAGAAATTTAT |
| 1 | DLBCHJT01 | ATTAGAACTAAATTATTCCATCACTAAAACT |
| 1 | DLBCHJT01 | ACTAAAACTAAATTATTCCATCACTAAAATT |
| 1 | DLBCHJT01 | ATTCTTAGCTATTCATAATTCATATTATTATAG |
| 1 | DLBCHJT01 | ATTATAGGCTATTCATAATTCATATTATTATAG |
| 1 | DLBCHJT01 | TTCTATTCTATATAGTTATTCTATATATAGAATAACTATATAGTATA |
| 1 | DLBCHJT01 | TATTCTATATAGTTATTCTATATATAGAATAACTATATAGTATAAAA |
| 1 | DLBCHJT01 | AATAAAAAATTGGATTAATGATCCAATTTTTTATT |
| 1 | NXXCHJT01 | TTTCTCTTTTTTCCCTTTTTAGAATTCTAA |
| 1 | NXXCHJT01 | TTAGAATTCTAAGAAGGGATGAAAGAGAAA |
| 1 | QPQWHJTM01 | TTTCGTTCTAATTTGTTCTTTTTTTTGTTCTAATTTGTTCTTTTTTTT |
| 1 | QPQWHJTM01 | TTTTGTTCTAATTTGTTCTTTTTTTTGTTCTAATTTGTTCTTTTTTTT |
| 1 | QPQWHJTM01 | TTGGTTTCGTTCTAATTTGTTCTTTTTTTT |
| 1 | QPQWHJTM01 | TTTTTTTTGTTCTAATTTGTTCTTTTTTTT |
| 1 | QPQWHJTM01 | TCATAAATATGAGTCAGATCATAAATATGAGTCAGA |
| 1 | QPQWHJTM01 | TTTAATATATATATATATATATATATTATA |
| 1 | QPXYHJT01 | AGAAAAATAACTGCAATAGCTAAATGATGATG |
| 1 | QPXYHJT01 | TAATATATATATAGATTTATATATATATAT |
| 1 | QPXYHJT01 | ATTTATATATATATATATATATATATATTA |
| 1 | SDHJT02 | GTTTTTATTGAATTCTTTTGTTCATATTGA |
| 1 | SDHJT02 | GTTCATATTGAATTCTTTTGTTCATTTTGA |
| 1 | SDHJT | TTCATTAAAATTTAAAAGAAGTGATTGGTT |
| 1 | SDHJT | TTGGTTAAAATTTAAAAGAAGTGATTGGAT |
| 1 | SLHJT | GTATATCTATATAGAAATAAATAGAATAGT |
| 1 | SLHJT | GAATAGTTATATAGAAATAAATAGAATAGT |
| 1 | SLHJT | TTATATATATATATTTATATATATATATAT |
| 1 | SLHJT | ATATATATATATTTATATATATATATATAT |
| 1 | SLHJT | TTCTATATATAAATTTATATATATATATATA |
| 1 | SLHJT | TTATATATATATATTTATATATATATATATA |
| 1 | SLHJT | TATTATATATATATATTTATATATATATATATAT |
| 1 | SLHJT | ATATATATATATTTATATATATATATATATTATA |
| 1 | SLHJT | TATATTATATATATATATTTATATATATATATATA |
| 1 | SLHJT | TATATATATATATTTATATATATATATATATTATA |
| 1 | SLHJT | ATATATTTCTATATATAAATTTATATATATA |
| 1 | SLHJT | TATATATATATATTTATATATATATATATAT |
| 1 | SLHJT | ATAAATTTATATATATATATATAAATTTAT |
| 1 | SLHJT | TATATATTTCTATATATAAATTTATATATA |
| 1 | SLHJT | TATATATAAATTTATATATATATATATAAA |
| 1 | TBXYHJT01 | AAAGCGAGAGAAACCCCATCCCTCTCTTTCCTTTTTTAGCCCCCATGTCGCCACACAGGGGGGACATCGGGACGTAAAAAAGG |
| 1 | TBXYHJT01 | TTCTTCAATCATTAGTATAGATTCTTCAATCATTAGTATAGA |
| 1 | XJDHHJT01 | GAAAATTCATTTTTTTTTTTTGAAACTGCT |
| 1 | XJDHHJT01 | GAAAATTCATTTTTTTTTTTTTGAAATGCT |
| 1 | XJDHHJT01 | TCTATTCTATAACTAAATATATATATTTATAGTTATA |
| 1 | XJDHHJT01 | TATGAAAGCATGTAAGAACTCAGGGGCACCTTCCCCCTC |
| 1 | XJDHHJT01 | AAATTTCTATATATATATATATATAAATTT |
| 1 | XJHEHJT01 | ATAAATTTCTATATATATATATAAATTTAT |
| 1 | XJHEHJT01 | TATATTTATATATATAAATTTCTATATATA |
| 1 | XJHEHJT01 | TATATATATAAATTTCTATATATATATATA |
| 1 | XJYCHJT01 | TATATTATAATTTATTTACTTTTCTATATTAAA |
| 1 | XJYCHJT01 | TATATTAAAATTTATTTACTTTTCTATATTAAA |
| 1 | XJYCHJT01 | TAAATATATATATTTATATATATATATTTA |
| 1 | XJYCHJT01 | AGATATTATATTATAATATATTATAATATCT |
| 1 | XJYCHJT01 | TAGAGTATAGAAACTAGATATATCTAGTTTCTATACTCTA |
| 1 | XMHJT | TTTAATATATATATTTTAATATATATATATT |
| 1 | XMHJT | TTTAATATATATATATTTATATATATATATT |
| 1 | XMHJT | ATTTTAATATATATATATTTATATATATATATTA |
| 1 | XMHJT | TAATATATATATATTTATATATATATATTATAAT |
| 1 | XMHJT | AATATATATATATTTATATATATATATTATA |
| 1 | XMHJT | TTAATATATATATTTTAATATATATATATT |
| 1 | XMHJT | AATATATATATTTTAATATATATATATTTA |
| 1 | YNHJT | AGAAATTTCTATATATATATATATAAATTT |
| 1 | YNHJT | AAATTTCTATATATATATATATAAATTTAT |

**Supplementary Table 6.** The location of SSRs in the chloroplast genomes of *Rhodiola*.

| ID | SSR | LSC | IR | SSC | Gene | IGS | CDS | Exon | Intron |
| --- | --- | --- | --- | --- | --- | --- | --- | --- | --- |
| BTHJT | 48 | 31 | 4 | 13 | 22 | 26 | 12 | 0 | 11 |
| CBHJT | 48 | 34 | 2 | 12 | 25 | 23 | 15 | 0 | 11 |
| CJHJT | 49 | 33 | 2 | 14 | 23 | 26 | 14 | 0 | 10 |
| CYHJT | 49 | 31 | 2 | 16 | 25 | 24 | 14 | 1 | 11 |
| DHHJT | 54 | 35 | 4 | 15 | 24 | 30 | 14 | 0 | 11 |
| DLBCHJT01 | 49 | 33 | 2 | 14 | 26 | 23 | 13 | 0 | 15 |
| DQZJHJT01 | 50 | 37 | 0 | 13 | 23 | 27 | 14 | 0 | 10 |
| DZCJHJT | 50 | 37 | 0 | 13 | 23 | 27 | 14 | 0 | 10 |
| NXXCHJT01 | 52 | 35 | 2 | 15 | 23 | 29 | 12 | 0 | 12 |
| QPQWHJTM01 | 51 | 37 | 0 | 14 | 22 | 29 | 13 | 0 | 10 |
| QPXYHJT01 | 52 | 33 | 4 | 15 | 27 | 25 | 16 | 0 | 12 |
| SDHJT02 | 53 | 34 | 6 | 13 | 23 | 30 | 13 | 0 | 11 |
| SDHJT | 51 | 36 | 4 | 11 | 25 | 26 | 14 | 0 | 12 |
| SLHJT | 57 | 41 | 0 | 16 | 25 | 32 | 15 | 0 | 11 |
| TBXCHJT01 | 53 | 37 | 2 | 14 | 21 | 32 | 11 | 0 | 11 |
| TBXYHJT01 | 53 | 36 | 2 | 15 | 27 | 26 | 15 | 0 | 13 |
| THCBHJT01 | 50 | 33 | 2 | 15 | 22 | 28 | 13 | 0 | 10 |
| THKYHJT01-1 | 50 | 33 | 2 | 15 | 22 | 28 | 13 | 0 | 10 |
| XJDHHJT01 | 53 | 36 | 2 | 15 | 25 | 28 | 13 | 0 | 13 |
| XJHEHJT01 | 53 | 40 | 0 | 13 | 27 | 26 | 15 | 0 | 13 |
| XJYCHJT01 | 54 | 35 | 4 | 15 | 26 | 28 | 17 | 0 | 10 |
| XMHJT | 55 | 42 | 0 | 13 | 25 | 30 | 15 | 0 | 11 |
| YNHJT | 58 | 40 | 4 | 14 | 26 | 32 | 13 | 0 | 14 |

**Supplementary Table 7.** The location of tandem repeats in the chloroplast genomes of *Rhodiola.*

| ID | Number of TRF | InGene | InIGS | InCDS | InExon | InIntron |
| --- | --- | --- | --- | --- | --- | --- |
| QPQWHJTM01 | 20 | 6 | 14 | 6 | 0 | 0 |
| XJHEHJT01 | 17 | 6 | 11 | 6 | 0 | 0 |
| NXXCHJT01 | 18 | 5 | 13 | 5 | 0 | 0 |
| XJYCHJT01 | 23 | 9 | 14 | 6 | 0 | 3 |
| SDHJT | 21 | 6 | 15 | 6 | 0 | 0 |
| XMHJT | 21 | 8 | 13 | 8 | 0 | 0 |
| SDHJT02 | 22 | 5 | 17 | 5 | 0 | 0 |
| YNHJT | 14 | 6 | 8 | 6 | 0 | 0 |
| SLHJT | 17 | 5 | 12 | 5 | 0 | 0 |
| TBXYHJT01 | 24 | 9 | 15 | 8 | 0 | 1 |
| THCBHJT01 | 26 | 8 | 18 | 6 | 0 | 2 |
| THKYHJT01-1 | 26 | 8 | 18 | 6 | 0 | 2 |
| BTHJT | 20 | 9 | 11 | 5 | 0 | 4 |
| TBXCHJT01 | 14 | 5 | 9 | 5 | 0 | 0 |
| CBHJT | 19 | 5 | 14 | 5 | 0 | 0 |
| QPXYHJT01 | 18 | 5 | 13 | 5 | 0 | 0 |
| CJHJT | 18 | 5 | 13 | 5 | 0 | 0 |
| CYHJT | 16 | 7 | 9 | 6 | 0 | 1 |
| DHHJT | 22 | 6 | 16 | 6 | 0 | 0 |
| DLBCHJT01 | 23 | 8 | 15 | 8 | 0 | 0 |
| DQZJHJT01 | 19 | 7 | 12 | 7 | 0 | 0 |
| DZCJHJT | 19 | 7 | 12 | 7 | 0 | 0 |
| XJDHHJT01 | 24 | 8 | 16 | 7 | 0 | 1 |

**Supplementary Table 8.** The location of repeat structures in the chloroplast genomes of *Rhodiola.*

| ID | total | direct | palindromic | InGene | InIGS | InCDS | InExon | InIntron |
| --- | --- | --- | --- | --- | --- | --- | --- | --- |
| BTHJT | 22 | 7 | 15 | 3.5 | 18.5 | 1.5 | 0 | 2 |
| CBHJT | 24 | 9 | 15 | 4.5 | 19.5 | 2.5 | 0 | 2 |
| CJHJT | 24 | 9 | 15 | 4.5 | 19.5 | 2.5 | 0 | 2 |
| CYHJT | 25 | 10 | 15 | 5.5 | 19.5 | 2.5 | 0 | 3 |
| DHHJT | 45 | 19 | 26 | 5 | 40 | 3 | 0 | 2 |
| DLBCHJT01 | 25 | 9 | 16 | 3.5 | 21.5 | 1.5 | 0 | 2 |
| DQZJHJT01 | 24 | 9 | 15 | 4.5 | 19.5 | 2.5 | 0 | 2 |
| DZCJHJT | 24 | 9 | 15 | 4.5 | 19.5 | 2.5 | 0 | 2 |
| NXXCHJT01 | 22 | 7 | 15 | 3.5 | 18.5 | 1.5 | 0 | 2 |
| QPQWHJTM01 | 24 | 11 | 13 | 3.5 | 20.5 | 2.5 | 0 | 1 |
| QPXYHJT01 | 25 | 9 | 16 | 4.5 | 20.5 | 2.5 | 0 | 2 |
| SDHJT02 | 22 | 8 | 14 | 3.5 | 18.5 | 1.5 | 0 | 2 |
| SDHJT | 23 | 8 | 15 | 4.5 | 18.5 | 2.5 | 0 | 2 |
| SLHJT | 32 | 12 | 20 | 4.5 | 27.5 | 2.5 | 0 | 2 |
| TBXCHJT01 | 21 | 7 | 14 | 3.5 | 17.5 | 1.5 | 0 | 2 |
| TBXYHJT01 | 29 | 12 | 17 | 4.5 | 24.5 | 2.5 | 0 | 2 |
| THCBHJT01 | 25 | 11 | 14 | 4.5 | 20.5 | 2.5 | 0 | 2 |
| THKYHJT01-1 | 25 | 11 | 14 | 4.5 | 20.5 | 2.5 | 0 | 2 |
| XJDHHJT01 | 27 | 11 | 16 | 5.5 | 21.5 | 2.5 | 0 | 3 |
| XJHEHJT01 | 27 | 9 | 18 | 4.5 | 22.5 | 2.5 | 0 | 2 |
| XJYCHJT01 | 23 | 9 | 14 | 4.5 | 18.5 | 2.5 | 0 | 2 |
| XMHJT | 28 | 10 | 18 | 4.5 | 23.5 | 2.5 | 0 | 2 |
| YNHJT | 17 | 5 | 12 | 4 | 13 | 2 | 0 | 2 |
